# Vitamin A and Retinoid Derivatives for Lung Cancer: A Systematic Review and Meta Analysis

**DOI:** 10.1371/journal.pone.0021107

**Published:** 2011-06-27

**Authors:** Heidi Fritz, Deborah Kennedy, Dean Fergusson, Rochelle Fernandes, Steve Doucette, Kieran Cooley, Andrew Seely, Stephen Sagar, Raimond Wong, Dugald Seely

**Affiliations:** 1 Department of Research and Epidemiology, The Canadian College of Naturopathic Medicine, Toronto, Ontario, Canada; 2 Leslie Dan Faculty of Pharmacy, The University of Toronto, Toronto, Ontario, Canada; 3 Laboratory Medicine and Pathobiology (LMP), The University of Toronto, Toronto, Ontario, Canada; 4 Clinical Epidemiology, Ottawa Hospital Research Institute, Ottawa, Ontario, Canada; 5 Department of Surgery, Ottawa Hospital Research Institute, Ottawa, Ontario, Canada; 6 Department of Medicine, Juravinski Cancer Centre, McMaster University, Hamilton, Ontario, Canada; Univesity of Texas Southwestern Medical Center at Dallas, United States of America

## Abstract

**Background:**

Despite reported antiproliferative activity of vitamin A and its common use for cancer, there is no comprehensive synthesis of its safety and efficacy in lung cancers. To address this issue we conducted a systematic review of the safety and efficacy of vitamin A for the treatment and prevention of lung cancers.

**Methods and Findings:**

Two independent reviewers searched six electronic databases from inception to July 2009 for clinical, observational, and preclinical evidence pertaining to the safety and efficacy of vitamin A and related retinoids for lung cancers. 248 studies were included for full review and analysis. Five RCTs assessed treatment of lung cancers, three assessed primary prevention, and three looked at secondary prevention of lung cancers. Five surrogate studies, 26 phase I/II, 32 observational, and 67 preclinical studies were also included. 107 studies were included for interactions between vitamin A and chemo- or radiation- therapy. Although some studies demonstrated benefits, there was insufficient evidence overall to support the use of vitamin A or related retinoids for the treatment or prevention of lung cancers. Retinyl palmitate combined with beta carotene increased risk of lung cancer in smokers in the large CARET trial. Pooling of three studies pertaining to treatment and three studies on secondary prevention revealed no significant effects on response rate, second primary tumor, recurrence, 5-year survival, and mortality. There was a small improvement in event free survival associated with vitamin A compared to controls, RR 1.24 (95% CI 1.13–1.35). The synthetic rexinoid bexarotene increased survival significantly among a subset of patients in two RCTs (p<0.014, <0.087).

**Conclusions:**

There is a lack of evidence to support the use of naturally occuring retinoids for the treatment and prevention of lung cancers. The rexinoid bexarotene may hold promise for use among a subset of patients, and deserves further study.

## Introduction

Lung cancers account for 12% of cancers globally and are the most common cancer type second only to non-melanoma skin cancer [1]. Lung cancers are the leading cause of cancer mortality, with an estimated 159,390 deaths in 2009 in the United States alone [1], [2]. Because of their prevalence, severity, and lack of effective treatment, it is crucial to find new interventions for the treatment and prevention of this disease.

Vitamin A is the generic term for a family of related compounds consisting of retinol and its derivatives, the retinoids [3]. Historically, natural retinoids have been used in the treatment of lung cancers since the late 70's when the first human trials were conducted [4], [5], [6], [7] on the basis of antiproliferative effects specific to epithelial tissues and tumors [8], [9], [10], [11]. The development of synthetic retinoids occurred subsequently in an attempt to retain vitamin A's antiproliferative effects specific to epithelial tissue while avoiding dose limiting side effects such as skin and liver toxicity [4], [12]. A recent survey of lung cancer patients found that 54% used complementary and alternative therapies, with vitamin combinations being the most common, at 17% [13]. Additionally, out of ten natural health products (NHPs) assessed during an initial scoping review of the literature for this project, vitamin A had the greatest volume and quality of evidence around its use in lung cancers.

The body obtains vitamin A from two sources: preformed vitamin A (retinol and retinal in the form of retinyl esters), and provitamin A carotenoids (beta carotene, alpha carotene, beta cryptoxanthin) [14], [15]. Preformed vitamin A is found in cod liver oil, butter, eggs, animal products, and fortified grains [14]. Provitamin A carotenoids are found in highly pigmented vegetables such as carrots, squash, yams, and green leafy vegetables [14]. Once in the body, retinol is ultimately converted into retinoic acid and its isoforms, collectively known as retinoids [14]. Vitamin A is responsible for the maintenance of epithelial tissues such as skin, mucus membranes, and lung tissue; it supports immune function; and plays a key role in mediating vision [16], [17].

Provitamin A carotenoids, particularly beta carotene, have also been studied for the prevention of lung cancers. Beta carotene acts in part through its conversion to vitamin A in the intestine and liver, however, it also independently possesses singlet oxygen quenching activity. It has been estimated that up to 88% of beta carotene is converted to retinyl esters in the intestinal wall, while up to 30% enters lymphatic circulation unchanged [18], [19], [20]. Beta carotene that reaches the liver undergoes further conversion to retinol, however a small percentage enters the blood unchanged [18]. The absorption and conversion of beta carotene to retinol is limited by several factors, including dose administered, with enzymatic saturation occurring at higher doses; the surrounding food matrix; the antagonistic effect of other carotenoids and vitamin A in the diet; and the vitamin A status of the individual [18], [21]. Beta carotene can correct vitamin A deficiency but it does not achieve the supraphysiological serum retinol levels attained by direct vitamin A supplementation. As such it does not possess the toxicity characteristic of high dose vitamin A supplementation; rather it results in orange discoloration of the skin [19]. At supra-repletion doses, the physiological activity of beta carotene beyond that of restoring vitamin A levels is likely due to its independent redox function or effects on cellular communication [22], [23]. Therefore, vitamin A may have a function in lung cancers distinct from that of beta carotene. For this reason, and since therapeutic use of vitamin A in lung cancers typically involves induction of supraphysiological serum levels, we only focused on the use of preformed vitamin A and retinoids for the purposes of this review.

Retinoid compounds consist of a basic structure of four isoprenoid units joined in a head-to-tail manner [24]. A large amount of variability exists between retinoids, and synthetic derivatives, which differ considerably in structure from the naturally occurring retinoids, have been developed in the hopes of avoiding some of the toxicity associated with the latter. Retinoids may be selective toward the two major retinoid receptors, retinoic acid receptor (RAR) and retinoid X receptor (RXR), or non selective pan-agonists [25]. First generation, or naturally occurring/physiological retinoids are derived from retinyl palmitate, C_36_H_60_O_2_, and include the isomers of retinoic acid such as 13-cis retinoic acid (13 CRA), 9-cis retinoic acid (9 CRA), and all-trans retinoic acid (ATRA) [14], [25]. Tretinoin (ATRA) and isotretinoin (13 CRA) are pharmaceutically produced versions of these. Alternately, *novel* synthetics that do not exist naturally have been designed for use in cancer (bexarotene and fenretinide) and other proliferative conditions (etretinate) [26], [27]. The two most common supplemental forms of vitamin A are retinyl palmitate and retinyl acetate [14]. **Table 1** outlines the classification of major retinoids and their primary clinical uses [16], [28], [29], [30], [31] ****.

**Table 1:** Retinoid Classification.

Common name	Generic name (Brand name if applicable)	Classification	Primary Use
*Physiological retinoids*			
*First Generation*			
All trans retinoic acid	tretinoin	classical* natural or synthetic; RAR agonist [29], [30]	Acute promyelocytic leukemia (APL) [5]
9 cis retinoic acid	alitretinoin	nonclassical† natural or synthetic; RAR + RXR agonist [29], [30]	Kaposi's sarcoma [30]
13 cis retinoinc acid	isotretinoin (Accutane)	classical natural or synthetic; RAR agonist [25]	Acne vulgaris [5]
*Synthetic retinoids*			
*Second Generation*			
Same as generic name	etretinate (Tegrison) OR acretin (Soriatane) its metabolite	synthetic, receptor dependent, aromatic retinoid	Psoriasis [27], [31], [32]
*Third Generation*			
Same as generic name	bexarotene (Targretin)	synthetic receptor dependent; RXR agonist (rexinoid) [25]	Cutaneous T-cell lymphoma [5], [28], [33]
4-HPR (N-4 hydroxyphenyl retinamide)	fenretinide	synthetic RAR agonist and receptor independent retinoid (atypical‡ retinoid) [30]	Breast cancer chemoprevention [30]

*Classical refers to retinoic acid receptor (RAR) agonism.

†**Nonclassical** refers to rexinoid receptor (RXR) agonism.

‡**Atypical** refers to receptor independent retinoids.

Retinoid molecules possess an antiproliferative effect at the cellular level via growth arrest signaling, promotion of differentiation, and induction of apoptosis [25]. Most retinoid activities are mediated by nuclear receptors: the retinoic acid receptor (RAR) and the retinoid X receptor (RXR), with subtypes α, ß, and γ [25]. These receptors function as ligand-regulated transcription factors that modulate gene expression patterns [32]. The exact mechanisms by which these receptors exert their downstream effect are complex and as yet incompletely elucidated, however they are thought to activate pathways that converge on G1 cell cycle arrest [28]. Retinoic acid has also been shown to downregulate markers of proliferation such as hTERT and cyclins D1 and 3 [33], [34], markers of DNA damage such as 8-oxo dGuo [35], and growth factors such as epidermal growth factor receptor (EGFR) and vascular endothelial growth factor (VEGF), potentially inhibiting tumor growth, angiogenesis, and metastasis [28], [36]. Tumor progression has been associated with decreased RARß expression, as a result it has also been proposed that retinoic acid inductible RARß may act directly as a tumor suppressor [25]. Retinoids are also thought to modulate additional targets such as reactive oxygen species, mitochondrial permeability, lipoxygenase, cyclooxygenase-2 (Cox-2), nuclear factor-kB, ubiquitination, tumor necrosis factor-α, c-Myc, Ap-1, and cell surface death receptors [37].

Preneoplastic and neoplastic diseases successfully treated with retinoids include oral leukoplakia, cervical dysplasia and xeroderma pigmentosum (premalignant), and acute promyelocytic leukemia (APL), a malignant condition characterized by altered signalling through both the promyelocyte leukemia protein (PML) and retinoic acid receptor α (RARα) [25], [38]. Modest but encouraging results have been found in the treatment of other cancer types including: head and neck cancer, cutaneous T-cell lymphoma, and neuroblastoma [39], [40], with increased survival (p<0.01) and reduced rates of second primary tumors (p = 0.005) demonstrated in some studies [41], [42]. The utilization of retinoids for the chemoprevention of lung cancers has been controversial, however, exemplified in the findings of the large CARET trial (Beta Carotene and Retinol Efficacy Trial) [43].

Given the range of vitamin A utilization and the possibility for harm in lung cancers, all levels of evidence, clinical and preclinical, were included in this synthesis of current knowledge pertaining to the safety and efficacy of vitamin A and its natural and synthetic derivatives for the treatment or prevention of lung cancers. We also assessed for potential interactions between Vitamin A and retinoids with chemotherapy and radiation treatment.

## Methods

### Search Strategy

We searched the following electronic databases for all levels of evidence pertaining to vitamin A and lung cancer: Pubmed, EMBASE, CINAHL, AltHealthWatch, the Cochrane Library, and the National Library of Science and Technology, from inception to the end of July 2009. We used a broad based MeSH and keyword approach combining clinical (lung cancer) and therapeutic (vitamin A) search terms. Separate searches were conducted independently by HF and DAK. After initial searches were completed, the scope of the review was expanded to include keywords associated with synthetic and naturally occurring retinoids. This additional search was conducted based on the premise that retinoids are derivatives of vitamin A that retain a similar mechanism of action while ostensibly posing less risk of toxicity and furthermore are used clinically. **Table S1** provides details on the search terms and strategy used to collect the records for screening from both searches employed.

A third search was conducted in Pubmed and EMBASE from inception to the end of October 2009 to identify articles pertaining to vitamin A and interactions with drugs used in lung cancer treatment and/or radiation therapy, irrespective of cancer type. Evidence of interactions were from clinical trials, observational studies, case reports, and preclinical studies where chemotherapy drugs and/or radiation therapy for lung cancers were assessed.

### Study Selection

Screening of studies was initially conducted based on title review. In the event of uncertainty, abstracts and/or full texts were also reviewed. Only English language publications were included. For inclusion, human trials had to assess the efficacy of natural or synthetic retinoids in lung cancers for the purposes of treatment, primary or secondary prevention, reduction of side effects and toxicities associated with chemo- or radiation- therapy, or assess for potential interactions with these therapies. Uncontrolled human trials were included, however they were evaluated separately, and in the absence of a comparator arm, determination of positive or negative outcome was based on the authors' interpretation of their findings (i.e. self assessed). Biomarker studies in humans were included if they examined endpoints directly related to lung cancer risk or pathogenesis. All types of lung cancers (SCLC, NSCLC, mesothelioma) were included.

In order to be included, observational studies had to employ an objective measure of preformed vitamin A status, such as serum, plasma, or lung tissue retinol levels, and had to examine the risk of lung cancers either prospectively or be conducted in patients with lung cancers comparing vitamin A status to patients without cancer. Due to the high possibility for confounding, studies examining dietary intake alone were excluded from analysis. Observational studies examining intake of supplemental vitamin A were also included.

For inclusion, preclinical studies had to be conducted in lung cancer models and examine either anticancer effects of vitamin A or related retinoids, or their interaction with conventional chemo- or radiation- therapy. Preclinical studies were categorized by results as positive, negative, neutral, or mixed. The term “positive” designates studies that found significant anticancer effects from at least one of the retinoids tested in models of lung cancers, alone or additively with other agents; “negative” designates studies that found significant pro-carcinogenic effects alone or in combination with other agents; and “neutral” designates studies that found no significant beneficial effect, nor any evidence of harm. In the absence of reported levels of significance, the authors' interpretation was used to guide classification. Studies examining surrogate markers were includedz only if the surrogates related directly to lung cancer risk or pathogenesis.

### Data Extraction and Quality Assessment

Data extraction forms were piloted using duplicate review to assess inter-researcher reliability. Upon completion of data extraction in duplicate for fifty percent of human level studies, there were no major inconsistencies, and further duplication of data extraction was found to be redundant. Extraction sheets were prepared partly based on the Consolidated Standards of Reporting Trials (CONSORT) statement, the Newcastle-Ottawa scale (NOS), and the Score for Assessment of Physical Experiments on Homeopathy (SAPEH) for clinical trials, observational studies, and preclinical studies, respectively [44], . We also extracted data on study design, patient characteristics, exposures or interventions, and outcomes. Randomized controlled trials were additionally assigned a quality score based on the Jadad criteria [47]. Interactions related articles were extracted similarly and analyzed for information specifically pertaining to interactions.

### Statistical Analysis

For randomized controlled trials, outcome data were pooled using random effects models weighted by the inverse variance in Comprehensive Meta-analysis Version 2, Biostat, Englewood, NJ, USA. Results are presented as risk ratios with 95% confidence intervals. Heterogeneity was assessed using the I^2^ statistic.

## Results

### Search Results

**Figure 1:**
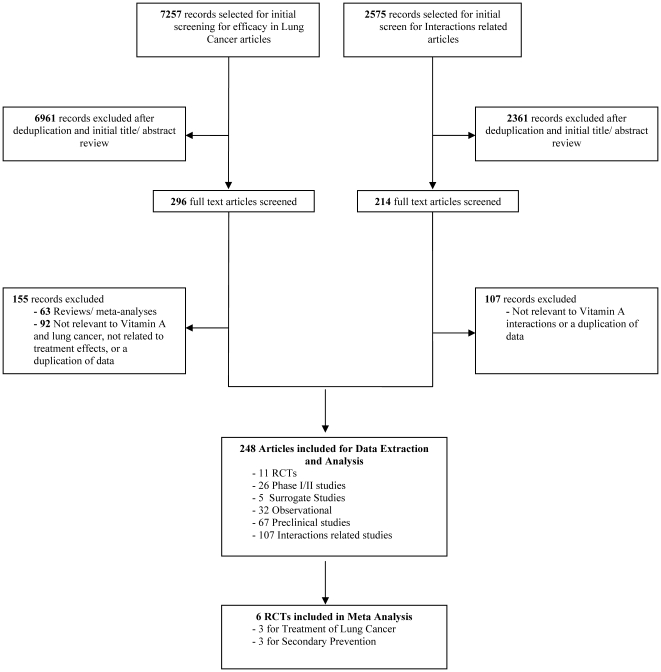
Flowchart of Literature Search and Study Selection.

The combined searches identified 7257 records for screening. A total of 141 studies were included for efficacy analysis. Five RCTs were included for the treatment of lung cancers, three RCTs looked at primary prevention, and three RCTs looked at secondary prevention of lung cancers. Five surrogate studies and 26 phase I/II studies were included. Thirty-two observational and 67 preclinical studies were also included. For the interactions component, 107 studies were included for full review and analysis. See **Figure 1** for a flowchart of studies included in the review.

### Preclinical Evidence

Sixty-seven preclinical studies of retinoids in lung cancer models were included for analysis. These studies were included in order to more clearly establish the biological rationale for retinoid use in lung cancers. Of the 67 studies, 54 showed results in favour of the retinoids tested, four showed mixed results, seven showed no effect, and two showed negative effects. Amongst the positive preclinical findings, the following anticancer effects were identified and synthesized: inhibition of growth and proliferation (n = 33 occurrences), proapoptotic (n = 15), chemopreventive (n = 14), anti-metastatic, anti-angiogenic, or anti-invasive (n = 7), lung cancer specific cytotoxicity (n = 3). Conversely, one study suggested a co-carcinogenic activity for supplemental retinyl palmitate when combined with 20-methylcholanthrene (20-MCA) [48] and one study showed a potential pro-angiogenic effect with increased VEGF levels [49]. Refer to **Table S2** for a detailed summary of preclinical evidence.

### Biomarker Trials

In humans, retinoids may modulate mediators of proliferation and some that are associated with lung cancer progression. An uncontrolled trial in early stage NSCLC patients suggested that bexarotene may modulate expression of biomarkers such as cyclins D1 and 3, and epidermal growth factor receptor (EGFR) [33], [50], [51], [52]. Of four trials investigating surrogate endpoints in high risk populations, two showed positive results including decreased expression of hTERT, a marker of proliferation, in bronchiolar tissue, and increased RARß expression (decreased expression of RARß has been associated with lung cancer development) [34], [53]. These trials used fenretinide and 13 CRA, respectively. Two of the four trials showed no significant effect on sputum atypia and bronchial cell metaplasia/dysplasia, also associated with lung cancer [54], [55]. Additionally, one observational study found no correlation between serum retinol and leukocyte 8-oxo-dGuo, a marker of DNA damage [35].

### Phase I/II Trials

Findings from the 26 included uncontrolled phase I or II trials suggest limited clinical benefit of vitamin A when used independently of chemotherapy. Of the 26 studies analyzed, 13 demonstrated positive results on response rates and/or survival time and 13 showed no significant effects (see **Table S3**). Of the positive trials, five used ATRA, four used 13 CRA, three used bexarotene, and two used retinol palmitate. Of the trials with no significant effect, five used 13 CRA, three used bexarotene, three used ATRA, one used 9 CRA and one used retinol; there were no major differences in dose ranges used between the studies with positive and negative findings. These phase I/II trials were conducted in patients with advanced stages of lung cancers, and in most cases, retinoids were used in conjunction with various chemotherapies including: docetaxel and capecitabine, cisplatin, etoposide, vindesine, mitomycin-C, carboplatin, gemcitabine, vinorelbine, 5-fluorouracil, interferon, and interleukin-2. Conversely, chemotherapy was rarely used in the trials showing no significant effects. None of the trials demonstrated disease exacerbation when vitamin A or retinoids were employed for treatment.

### Controlled Observational Studies

Thirty-two controlled observational studies were included for analysis. Seven of the studies were prospective, 16 were retrospective, and 9 were cross-sectional. Of all the observational studies included, nearly half (n = 15) found an inverse relationship between serum retinol levels and risk of lung cancers (longitudinal studies) and/or current diagnoses of lung cancers (cross-sectional studies). See **Tables S4** and **S5**.

### Controlled Clinical Trials

#### Treatment

**Figure 2:**
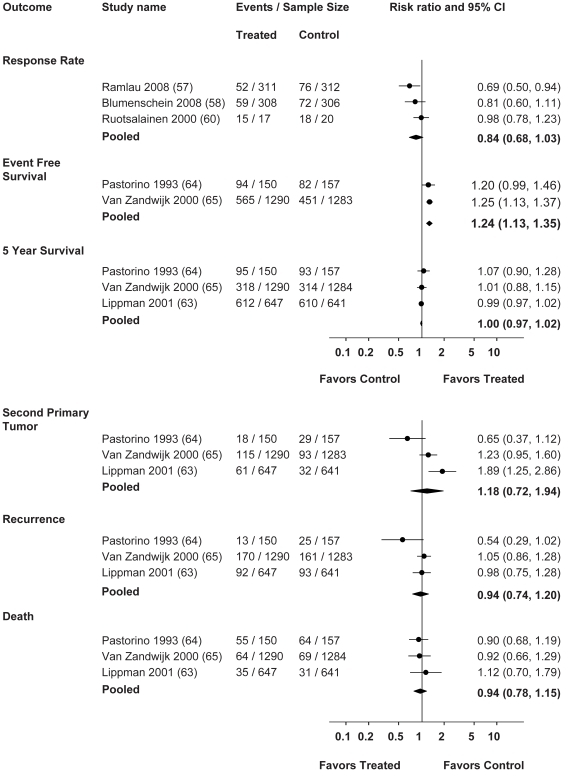
Forest Plots: Pooled Results of RCTs for the Treatment or Prevention of Lung Cancer.

Five interventional RCTs tested retinoids (bexarotene and 13 CRA) in either small cell or non-small cell lung cancers (see **Tables S4 and S5**) [56], [57], [58], [59], [60]. Of these, one trial comparing 13 CRA + IL-2 versus IL-2 alone demonstrated improvements in immunological parameters and a decrease in VEGF. In this trial, a trend towards improvement was also seen in progression free survival (PFS): 39.3% in the treatment arm versus 30.4% of controls were progression free at 42 months, with a median PFS of 28.45 months versus 12.35 months, treatment versus control (HR 0.7356, 95% CI 0.46–1.163, p = 0.185) [56]. Meta analysis of three trials measuring response rates found no significant effect overall, relative risk RR 0.84, (95% CI 0.68–1.03, I^2^ = 40.4%). For one study that reported response rates in terms of complete response, partial response, progressive disease, or disease stabilization, the response rate was calculated based on the number of complete and partial responses [60]. See **Figure 2**.

Two RCTs examining adjuvant bexarotene therapy found significant survival benefits among a subset of patients who experienced Grade 3/4 hypertriglyceridemia (≥5x upper limit of normal), compared to the control group: Ramlau reported that these patients had median survival of 12.3 mo compared to 9.9 mo in the control group (p = 0.087), while Blumenschein reported median survival of 12.4 mo compared to 9.2 mo in similar patients (p = 0.014) [57], [58]. These responsive patients that represented approximately 30–40% of the treatment group also had a higher incidence of side effects including skin rash and hypothyroidism, suggesting that hypertriglyceridemia may be a surrogate of greater systemic biochemical sensitivity to the effects of bexarotene [57]. Conversely, of the patients receiving bexarotene and who did not experience Grade 3/4 hypertriglyceridemia, a worse treatment response was reported, with significantly shorter survival than the placebo groups (p<0.0001 for both), highlighting the need to efficiently identify responders [57], [58], Rizvi also examined bexarotene, however the study was prematurely terminated due to poor enrollment [59].

#### Primary Prevention

Three controlled clinical trials were found pertaining to vitamin A and primary prevention of lung cancers [43], [61], [62]. All of the clinical trials used retinyl palmitate. Two trials showed no significant effects on lung cancer prevention overall [61], [62], however, one of these did find a significantly decreased risk of mesothelioma only as separate from SCLC and NSCLC, RR 0.24 (95% CI 0.07–0.86) [61]. These trials were not pooled due to heterogeneity in the reporting of results. The large Carotene and Retinol Efficacy Trial (CARET), however, found significant increases in lung cancer incidence from combined treatment with retinyl palmitate and beta carotene when administered in high risk populations. Trial results demonstrated an increase in overall lung cancers, (RR 1.28; 95% CI 1.04-1.57). Subgroup analysis showed higher risk in asbestos workers (RR 1.40, 95% CI: 0.95–2.07), and current heavy smokers (RR 1.42, 95% CI: 1.07–1.87), while finding a non significant reduction in risk amongst smokers who had already quit at randomization (RR 0.80, 95% CI: 0.48–1.31) [43]. Relative risk of death from all causes was 1.18 (95% CI: 1.02–1.37), death from lung cancers 1.46 (95% CI 1.07–2.00), and death from cardiovascular disease 1.26 (95% CI 0.99–1.61) [43]. There was no evidence of increased risk of other cancer types.

#### Secondary Prevention

Three controlled trials for the secondary prevention of lung cancers were included [63], [64], [65]. Lippman found no significant effects overall, however, lung cancer recurrence and all cause mortality were significantly increased in current smokers receiving isotretinoin compared to placebo, HR 3.11 (95% CI 1.00–9.71) and 4.39 (1.11–17.29) respectively [63]. Meta analysis of these three RCTs for secondary prevention showed no significant effects for vitamin A on second primary tumor (RR 1.18, 95% CI 0.72–1.94, I^2^ = 78.7%), recurrence (RR 0.94, 95% CI 0.74–1.20, I^2^ = 47.1%), 5-year survival (RR 1.00, 95% 0.97–1.02, I^2^ = 0%), and death (RR 0.94, 95% CI 0.78–1.15, I^2^ = 0%). There was a small but significant improvement in event free survival associated with vitamin A compared to controls, RR 1.24 (95% CI 1.13–1.35, I^2^ = 0%), based on data from two RCTs. See **Figure 2**.

### Safety and Risks Associated with Use

#### Adverse Events

In controlled clinical trials, the most common side effects observed were grade I-II mucocutaneous symptoms symptoms including dry skin, rash, conjunctivitis, and chelitis, reversible hypothyroidism, and hyperlipidemia, especially hypertriglyceridemia. In the two controlled studies of bexarotene that reported incidence, between 63–66% of treated patients experienced hypertriglyceridemia compared to 1.3–2.0% of control groups, and between 12–25% experienced hypothyroidism compared to 0.3–0.7% of the control groups [58], [60]. Hypertriglyceridemia was usually manageable with antilipid therapy, however in some cases dose reduction was necessary to prevent patient withdrawal from the study due to toxicity (up to 18%) [58]. Up to 24% of patients treated with bexarotene developed hypothyroidism manageable by thyroid hormone replacement therapy [58].

In the Phase I/II trials, the most common side effects observed were mucocutaneous symptoms (occurring amongst most patients in some of the trials); hyperlipidemia, in particular hypertriglyceridemia, ranging from under 44 to 86% in frequency; and elevated liver function tests (LFTs), in up to 54%. Hypertriglyceridemia and elevated liver enzymes were most often a grade I/II severity, but reached grade III/IV in some cases; hypertriglyceridemia and elevated liver enzymes were reversible upon dose reduction or cessation. Less common side effects included mild gastrointestinal symptoms, headaches, myalgia/arthralgia, and fatigue. Hematological toxicities occurred in trials combining retinoids with chemotherapy, however, these were not common in trials of retinoids alone, suggesting chemotherapy as the causative agent.

#### Dose Limiting Toxicities

In a Phase I trial of ATRA, dose limiting toxicities (DLTs) of mucocutaneous symptoms, headache, fatigue, gastrointestinal symptoms, myalgia, and confusion, were established. These DLTs occurred at 150 mg/m^2^ ATRA in conjunction with cisplatin and etoposide [66]. In a phase I trial of bexarotene, DLTs included one case of a pain syndrome and one case of increased creatinine kinase (CK), however the maximum tolerated dose (MTD) was not reached [67]. Yet another phase I trial using escalating doses of 200, 300, and 400 mg/m^2^ bexarotene did not reach a MTD as defined at outset, which was the dose level immediately below the dose level at which 2 patients of a dose level experienced DLT during their first cycle [68].

### Interactions

**Table S6** provides an overview of the effects of retinoid/chemotherapeutic drug combinations in specific cell lines *in vitro*. The effects summarized include a combination index (CI) between the two substances, if reported, and when no CI was reported an account of either a positive or negative impact on neoplastic cell growth from the combination is provided in the table.

Emphasis is placed here on the pharmacokinetics changes and adverse events reported in the literature of the various vitamin A forms and the chemotherapy drugs used in the treatment of lung cancer. Case reports were also reviewed however, no relevant information could be garnered on interactions and these did not enhance the results, and thus are not reported.

### Fenretinide

One phase I study found a reduced C_max_ and AUC on day 8 for fenretinide in combination with cisplatin and paclitaxel, however, in the absence of serum data, it was not possible to determine whether the reduction was due to reduced absorption or increased elimination kinetics [69]. Significant nyctalopia (night blindness), causing treatment delays, was also reported with the combination of fenretinide/cisplatin/paclitaxel. Nyctalopia has also been reported with isotretinion and is thought to develop due to hypovitaminosis A, which results in a reduction in rhodopsin for the photoreceptors [70].

### Bexarotene

Dose limiting toxicities for bexarotene have not been reported up to 500 mg/m^2^, however, at 600 mg/m^2^ and above cases of hypertriglycemidemia leading to acute pancreatitis have been reported [71].

### All-trans retinoic acid (ATRA)

A larger body of research has been conducted regarding the pharmacokinetics of ATRA. Absorption of oral ATRA is variable and highly dependent upon a high fat content in the intestinal lumen, as ATRA is almost insoluble in an aqueous environment [72]. Continuous dosing of ATRA has been found to accelerate elimination of ATRA and an “on-off” the dosing schedule is suggested to avoid the increased elimination [72]. ATRA has been found to induce Retinoic Acid Syndrome (RAS) in APL and AML patients, which can be fatal in some.

### 13*cis* – retinoic acid (13CRA)

Waladkhani and Clemens studied the pharmacokinetics of 13 CRA along with interferon α in cancer patients. Their results demonstrated an increase conversion to the less potent metabolite, 4-oxo 13CRA, in cancer patients versus healthy volunteers possibly through the up regulation of CYP induced by interferon α [73]. 13CRA was found to decrease elimination of paclitaxel and increase the AUC of the metabolite 6-alfa-hydroxytaxol [74].

## Discussion

Although vitamin A and related retinoids have been widely used for the treatment and prevention of lung cancers, our review suggests that there is a lack of evidence to support this use. Preliminary evidence including preclinical and observational studies has shown promising results with respect to lung cancer pathogenesis and disease risk, respectively, however these effects have not translated to human interventional settings. Encouraging preliminary results were found by Pastorino et al. in a setting of secondary prevention of NSCLC following surgical resection as well as by de Klerk et al in primary prevention of mesothelioma, but these have not been replicated by other groups [61], [64]. The CARET trial found adverse effects on overall lung cancer risk among smokers and asbestos workers, and this seems to be supported by similar effects among current smokers in the trial by Lippman [43], [63].

There is a lack of RCTs investigating retinoids for lung cancer treatment; a single trial exists showing clinical benefit when 13 CRA, a naturally occurring retinoid, was combined IL-2, and this on surrogate immune parameters and levels of VEGF only. Two RCTs investigating the synthetic rexinoid bexarotene have shown significant survival benefits in a third of patients manifesting hypertriglyceridemia as a surrogate of sensitivity, but worse outcomes in non-responders [57], [58]. Further research is required to elucidate the best genetic predictors or biochemical surrogates of responsiveness and to confirm these post hoc findings before bexarotene can be recommended for wider clinical use [75]. At present these results should not be extrapolated to other retinoids due to the considerable biochemical differences between them, however, any future research investigating retinoids, classical or not, should adopt a similar strategy of identifying a surrogate of responsiveness among a subgroup of patients.

The reasons for the observed divergence and the lack of applicability between preclinical findings and the clinical setting are not clear. It is possible that the appropriate subset of patients who might benefit from retinyl palmitate or other retinoids has not been adequately identified by the clinical research to date, as has been done in the case of bexarotene. Alternately, this divergence may arise from the inherent differences between preclinical and clinical research. One theory has been suggested that preclinical lung cancer models induced by exposure to single carcinogens such as the nitrosamine 4-(methylnitrosamino)-1-(3-pyridyl)-1-butanone (NNK) do not accurately mimic the effects of a complex carcinogen such as cigarette smoke, well accepted to be the most common cause of lung cancer [76]. This inconsistency has been confirmed, for instance, in studies of selenium comparing chemoprevention in both complex- or single- carcinogen lung cancer models, wherein selenium was able to prevent lung tumors induced by NNK, but not those caused by exposure to tobacco smoke [76], [77].

With respect to observational evidence, it is possible that serum retinol may be a biomarker of other active anticancer substances in the diet or of an overall “healthy” dietary pattern. In this case, interventions with vitamin A alone would be expected to fail. The biomarker theory is even more applicable to beta carotene which correlates more directly than serum retinol with intake of fruits and vegetables and what is considered an overall health-promoting diet rich in a complex array of chemopreventive substances such as flavonoids and isothiocyanates [78], [79]. This biomarker hypothesis may explain in part the failure of vitamin A as an interventional agent, and is especially true insofar as beta carotene contributes to normal (but not supraphysiological) vitamin A status.

Importantly, CARET, the largest chemoprevention trial to date of vitamin A and lung cancers, found a significantly increased risk of lung cancers in the retinol and beta carotene arm [43]. This trial, consisting of 18,314 current and former smokers and asbestos workers at high risk of lung cancers, was stopped early due to interim results showing increased lung cancer risk in the active arm (retinol and beta carotene), RR 1.28 (1.04–1.57) [43]. When reported according to the prespecified weighted analysis, this increased to RR 1.36 (95% CI 1.07 – 1.73) [80]. Follow up results found that the risk of lung cancers and overall risk of death were elevated up to six years after the intervention was discontinued, with greatest impact occurring in women [81]. Adjustment for baseline beta carotene levels did not modify the elevated risks associated with active treatment, and the effect retinol and beta carotene was greatest several years after beginning consumption [80].

A biomarker study was conducted in a small subgroup of the CARET population to determine the effect of supplementation on target tissue levels: bronchoalveolar lavage showed that while tissue levels of beta carotene increased significantly with supplementation, retinol levels did not [82]; serum levels increased for both [83]. This suggests that beta carotene, independent of its provitamin A activity, may be the culprit responsible for the deleterious effect on lung cancers observed in this trial. This hypothesis is supported by similar findings from the large Finnish Alpha Tocopherol Beta Carotene (ATBC) trial, conducted in 29,133 male smokers for 5 to 8 years. Investigators found that supplementation of 20 mg beta carotene without vitamin A increased incidence of lung cancers by 16% compared to those not receiving beta carotene, RR 1.16 (95% CI 1.02 – 1.33) [84]. Serum retinol levels rose only 6% compared to placebo in those receiving beta carotene supplementation (p = 0.03) [85]. This increase in risk is roughly comparable to the 28% effect seen in CARET.

While the mechanism for the detrimental effects observed have not been fully elucidated, it is hypothesized that under conditions of high oxidative stress and exposure to lung irritants such as those associated with exposure to cigarette smoke and asbestos, certain antioxidants may in fact act as conditional pro-oxidants [22], [86], [87], [88]. According to the conditional pro-oxidant hypothesis, the activity of an antioxidant depends on its redox potential in relation to other pro- and anti- oxidants in its microenvironment. Carotenoids are particularly vulnerable to such oxidation due to their long chains of conjugated double bonds, and are known to concentrate in the lungs [89], [90], [91]. New evidence suggests that carotene breakdown products (CBPs) produced by high dose administration under conditions of oxidative stress may act as pro-oxidants, impairing mitochondrial function and resulting in cellular damage, thereby predisposing to carcinogenesis [22], [91].

Limitations of the chemoprevention studies listed here include lack of a single-agent intervention arm in CARET, and lack of a placebo arm in the Western Perth study to distinguish the potentially differing effects of beta carotene and retinol. CARET also used a synthetic form of beta carotene. As discussed above, while beta carotene is a precursor to retinol in the body, it is nonetheless physiologically distinct in its own right, possessing redox activity independent of vitamin A [90]. Also, while CBPs have been shown to impair mitochondrial function, preliminary evidence suggests that retinol may be an essential response modifier and cofactor of mitochondrial energy production [92]. The limitations of these chemoprevention trials, however, impede our ability to draw any definite conclusions about the differential effects of beta carotene and retinoids on lung cancers in humans.

Notably, a 2008 systematic review and meta- analysis focused exclusively on beta carotene in relation to cancer risk. Investigators screened 22,994 records and included six RCTs plus 30 prospective observational studies [223]. The RCTs included in this review, three of which have already been described above, were: 1) the Carotene and Retinol Efficacy Trial (CARET) [43]; 2) the Alpha Tocopherol Beta Carotene (ATBC) study [94]; 3) the Physicians' Health Study (PHS) [95]; 4) the Western Perth, Australia study [61]; 5) the Women's Health Study [96]; and 6) the Linxian General Population trial [62]. The pooled RR for studies comparing beta carotene supplements to placebo (n = 3) was 1.10 (95% CI 0.89 – 1.36) [93]. In trials conducted in high risk populations comprised of smokers and asbestos workers (n = 2), there were significant increases in lung cancer risk associated with beta carotene supplementation: in the ATBC trial, RR 1.17 (95% CI 1.02 – 1.34), and in the CARET trial 1.36 (1.07 – 1.72) [93]. In contrast to this interventional data but in agreement with the biomarker hypothesis, analysis of observational data found that the pooled RR for lung cancers was decreased in the highest versus the lowest category of total dietary carotenoid intake for studies reporting smoking-adjusted risk was 0.79 (95% CI 0.71 – 0.87) [93]. For intake of beta carotene specifically, there was a non-significant 8% reduction in smoking-adjusted risk, RR 0.92 (95% CI 0.83 – 1.01) [93].

This analysis is limited by an exclusive focus on lung cancers and as such we are unable to draw any conclusions about the application of retinoids in other cancer types. It is important to note that there is possible benefits from retinoids in conditions such as head and neck cancer, mesothelioma, and certain premalignant conditions. ATRA combined with chemotherapy has been successful in treating acute promyelocytic leukemia (APL), inducing remission in what was once a highly lethal leukemia [25], [97]. Reports have shown that classical retinoids including 13CRA and ATRA may be effective for treatment of premalignant lesions including leukoplakia, actinic keratosis, and cervical dysplasia [25], [40], [98]. In controlled trials on prevention of head and neck cancer, retinyl palmitate, 13 CRA, and fenretinide have demonstrated significant response rates [40], [99].

With respect to mesothelioma, cancer of the pleural lining of the lungs, one of the studies reviewed here under primary prevention found a significantly decreased risk of mesothelioma in the retinol group, RR 0.24 (0.07-0.86) [61]. A limitation of this study was the lack of a placebo comparator arm; retinol was instead compared to beta carotene. It is possible that beta carotene had an overall adverse effect on disease risk, thereby producing a falsely positive effect for retinol in comparison, however, this is impossible to determine.

Strengths of this analysis include a comprehensive and systematic survey of the literature with a clear focus on lung cancers. All levels of evidence, human, observational, and preclinical were included to achieve a broad analysis of anticancer activity of vitamin A/retinoids in lung cancers, and any possible interactions with chemotherapy and/or radiation therapy to assess safety alongside efficacy. We are not aware of another review of vitamin A for lung cancers to date that has included such an extensive analysis of the available data.

## Supporting Information

Table S1 Search Strings for Vitamin A and Lung Cancer. *Note: “Lung neoplasm” was the MESH term used in Pubmed; in other databases, “Lung cancer” was used.
(DOC)

Table S2 **Preclinical Evidence.** 1,25 vitD 1,25 dihydroxyvitaminD; 4-HPR fenretinide; 9CRA 9 cis retinoic acid; 13CRA 13 cis retinoic acid; ATRA all trans retinoic acid; Bex bexarotene; CDDP cisplatin; d/o depends on; GCB gemcitabine; MDR multi drug resistance; NGF nerve growth factor; NSCLC non samll cell lung cancer; PAX paclitaxel; RRMs retinoid relateed molecules; SCLC small cell lung cancer; VIN vinorelbine; w/w/o with or without. **Footnotes:** *Includes effects on tumor growth observed in animal models of metastasis to the lungs, eg., such as that induced by intravenous or subcutaneous injection of lung cancer cells, as well as results from in vitro on markers such as VEGF or invasive capacity. Thus results from animal models of metastasis are differentiated from effects on primary tumor growth induced by adniminstration of carcinogen, and are detailed under this column, whereas measures of (non-metastatic) primary tumor growth are categorized under “Anticancer Effect”. † + results in favour of vitamin A; - detrimental results found with vitamin A use; m mixed effects both positive and negative; n no significant effect or neutral result; y yes effect demonstrated; – not applicable/outcome not assessed. ‡ “Vitamin A” stated but precise form not specified.
(DOC)

Table S3 **Uncontrolled Phase I/II Trials for the Treatment of Lung Cancer in Order of the Form of Vitamin A.** 5-FU 5-fluorouracil; adv advanced; bw between; CK: creatinine phosphokinase; CRA cis retinoic acid; d day; DLTs dose limiting toxicities; EFS event free survival; Gr Grade; IFN interferon; LFT's liver function tests; MIU =  million IU; mo month; NS =  non significant; NSCLC non small cell lung carcinoma; OS overall survival; PFS progression free survival; PHA phytohemagglutinin; pt patients; resp respectively; sign significant; SC subcutaneous; SCC small cell carcinoma; TC total cholesterol; TG triglyceride; TTP time to progression; w with; VEGF vascular endothelial growth factor; wk week; yr year.
(DOC)

Table S4 **Methodologies of Controlled Human Studies for Vitamin A and Lung Cancer.** CA cancer; CAD coronary artery disease; comb combination; f/u follow up; GI gastrointestinal; LTFU loss to follow up; NR not reported; NSCLC non small cell lung cancer; pop population; PS performance score; SCLC small cell lung cancer; w with.
(DOC)

Table S5 **Outcomes of Controlled Human Studies for Vitamin A and Lung Cancer.** 95% CI 95% confidence interval; Ad adenocarcinoma; Adj adjusted; AHR adjusted hazard ratio; AOR adjusted odds ratio; C control group; CR complete response; DS disease stabilization; DSR disease stabilization rate; EC epidermoid carcinoma; HR hazard ratio; LC large cell carcinoma; mo months; NR not reported; NS not significant; OR odds ratio; PD progressive disease; PR partial response; RR relative risk; SmC small cell carcinoma; T treatment group.
(DOC)

Table S6 **Combination Effect,** ***in vitro*** **, Between Forms of Vitamin A and Chemotherapy Drugs.**
(DOC)
