# Supplementary material for: Vitamin A and Retinoid Derivatives for Lung Cancer: A Systematic Review and Meta Analysis
Source: PLoS One. 2011 Jun 27;6(6):e21107. doi: 10.1371/journal.pone.0021107 (PMC3124481; doi:10.1371/journal.pone.0021107)
Supplement: Table S1 — Search Strings for Vitamin A and Lung Cancer. *Note: “Lung neoplasm” was the MESH term used in Pubmed; in other databases, “Lung cancer” was used. (DOC) [file pone.0021107.s001.doc]

**Table S**1. Search Strings for Vitamin A and Lung Cancer

| **Search Strings AND** | |
| --- | --- |
| Vitamin A | Lung Neoplasm* |
| Retinoic acid | Lung Neoplasm |
| Retinol | Lung Neoplasm |
| Vitamin A | Lung Neoplasm AND Chemoprevention |
| Retinoids | Lung Neoplasm |
| Bexarotene OR Fenretinide | Lung Neoplasm or Chemoprevention |
