# Supplementary material for: Vitamin A and Retinoid Derivatives for Lung Cancer: A Systematic Review and Meta Analysis
Source: PLoS One. 2011 Jun 27;6(6):e21107. doi: 10.1371/journal.pone.0021107 (PMC3124481; doi:10.1371/journal.pone.0021107)
Supplement: Table S2 — Preclinical Evidence. 1,25 vitD 1,25 dihydroxyvitaminD; 4-HPR fenretinide; 9CRA 9 cis retinoic acid; 13CRA 13 cis retinoic acid; ATRA all trans retinoic acid; Bex bexarotene; CDDP cisplatin; d/o depends on; GCB gemcitabine; MDR multi drug resistance; NGF nerve growth factor; NSCLC non samll cell lung cancer; PAX paclitaxel; RRMs retinoid relateed molecules; SCLC small cell lung cancer; VIN vinorelbine; w/w/o with or without. Footnotes: *Includes effects on tumor growth observed in animal models of metastasis to the lungs, eg., such as that induced by intravenous or subcutaneous injection of lung cancer cells, as well as results from in vitro on markers such as VEGF or invasive capacity. Thus results from animal models of metastasis are differentiated from effects on primary tumor growth induced by adniminstration of carcinogen, and are detailed under this column, whereas measures of (non-metastatic) primary tumor growth are categorized under “Anticancer Effect”. † + results in favour of vitamin A; - detrimental results found with vitamin A use; m mixed effects both positive and negative; n no significant effect or neutral result; y yes effect demonstrated; – not applicable/outcome not assessed. ‡ “Vitamin A” stated but precise form not specified. (DOC) [file pone.0021107.s002.doc]

**Table S2. Preclinical Evidence**

| **DESIGN** | | | | | | | **OUTCOMES MEASURED & EFFECTS** | | | | | | | | |
| --- | --- | --- | --- | --- | --- | --- | --- | --- | --- | --- | --- | --- | --- | --- | --- |
| Ref. | In vitro | In vivo | Ex vivo | N | Retinoid | Chemo-therapy | Anticancer Effect (↓Lung tumor incidence/ multiplicity/ volume) | ↑Survival/ ↓Mortality/ ↑time to tumor development | Antiproliferative effect/ growth inhibition | Proapoptotic effect | Anti-metastatic/ angiogenic/ invasive effect***** | Pro-carcinogenic/ pro-angiogenic | Impact on drug effect (↑ or ↓) | **Other** | **+/-n/m†** |
| Kadara 2008 [1] | y | -- | -- | -- | 4-HPR | n | -- | -- | -- | y | -- | -- | -- | Rac activation | + |
| Schroeder 2006 [2] | y | -- | -- | -- | 4-HPR | n | -- | -- | y | y | -- | -- | -- | -- | + |
| Sun 2005 [3] | y | -- | -- | -- | 4-HPR | celecoxib | -- | -- | y | y | -- | -- | ↑ | -- | + |
| Ohlmann 2002 [4] | y | -- | -- | -- | 4-HPR, ATRA | n | -- | -- | y | y | -- | -- | -- | -- | + |
| Sun 2001 [5] | y | -- | -- | -- | 4-HPR and 6 other retinamides | n | -- | -- | y | n | -- | -- | -- | -- | + |
| Maurer 2000 [6] | y | -- | -- | -- | 4-HPR | n | -- | -- | y | -- | -- | -- | -- | Cytotoxicity | + |
| Kalemkerian1999 [7] | y | -- | -- | -- | 4-HPR | etoposide, PAX, CDDP | -- | -- | y | -- | -- | -- | ↑ | Cytotoxicity | + |
| Zou 1998 [8] | y | -- | -- | -- | 4-HPR, ATRA | n | -- | -- | y | y | -- | -- | -- | -- | + |
| Kalemkerian 1995 [9] | y | -- | -- | -- | 4-HPR, ATRA | n | -- | -- | y | y | -- | -- | -- | -- | + |
| Choi 2007 [10] | y | -- | -- | -- | ATRA, 13CRA, 9CRA, 4-HPR | n | -- | -- | y | -- | -- | -- | -- | synergy between 4-HPR and ATRA or 13CRA | + |
| Kadara 2006 [11] | y | -- | -- | -- | ATRA, 4-HPR, 3 RRMs | n | -- | -- | -- | y | -- | -- | -- | ↑ p53 | + |
| Kawakami 2006 [12] | y | -- | -- | -- | ATRA (liposomal) | n | -- | -- | -- | y | -- | -- | -- | Cytotoxicity | + |
| Tabata 2006 [13] | y | y | -- | NR | ATRA | γ-rays | -- | -- | y | -- | -- | -- | -- | Protection against radiotherapy induced pulmonary injury | + |
| Suzuki 2006 [14] | -- | y | -- | NR | ATRA (liposomal) | n | -- | -- | -- | -- | y | -- | -- | distribution high within lung tissue | + |
| Lin 2005 [15] | y | -- | -- | -- | ATRA | w/w/o As2O3 | -- | -- | y | y | -- | -- | synergy | ATRA ↑proapoptotic effect of As2O3 | + |
| Ma 2005 [16] | y | -- | -- | -- | ATRA | n | -- | -- | -- | -- | -- | -- | -- | ↓carcinogenic transformation via ↓D1 and D2 cyclin expression | + |
| Maeno 2002 [17] | y | -- | -- | -- | ATRA | n | -- | -- | -- | -- | -- | y | -- | -- | - |
| Albright 2002 [18] | y | -- | -- | -- | ATRA | n | -- | -- | y | -- | -- | -- | -- | ↑ differentiation | + |
| Fiorentini 2002 [19] | y | y | -- | 20 | ATRA | NGF | y | -- | n | -- | y | -- | -- | No direct effect of ATRA alone on tumor size, but prevented ↑in tumor size induced by NGF withdrawal | + |
| Hsu 2000 [20] | y | -- | -- | -- | ATRA | n | -- | -- | y | -- | -- | -- | -- | -- | + |
| Manna 2000 [21] | y | -- | -- | -- | ATRA | n | -- | -- | -- | y | -- | -- | -- | ↑ TNF | + |
| Agadir 1999 [22] | y | -- | -- | -- | ATRA | n | -- | -- | y | -- | -- | -- | -- | ↓ AP-1 | + |
| Lokshin 1999 [23] | y | -- | -- | -- | ATRA | n | -- | -- | y | y | -- | -- | -- | -- | + |
| Weber 1999 [24] | y | -- | -- | -- | ATRA | n | -- | -- | y | n | -- | -- | -- | -- | + |
| Li 1998 [25] | y | -- | -- | -- | ATRA | n | -- | -- | y | y | -- | -- | -- | -- | + |
| Liu 1998 [26] | y | -- | -- | -- | ATRA, 4-HPR | n | -- | -- | y | -- | -- | -- | -- | -- | + |
| Guzey 1998 [27] | y | -- | -- | -- | ATRA, 9CRA | n | -- | -- | y | y | -- | -- | -- | -- | + |
| Ross 1996 [28] | y | -- | -- | -- | ATRA | n | -- | -- | y | -- | -- | -- | -- | -- | + |
| Higashimoto 1996 [29] | y | -- | -- | -- | ATRA | n | -- | -- | y | -- | -- | -- | -- | -- | + |
| Kalemkerian 1994 [30] | y | -- | -- | -- | ATRA | n | -- | -- | -- | -- | -- | -- | -- | Prevention of SCLC transformation to NSCLC phenotype | + |
| Geradts 1993 [31] | y | -- | -- | -- | ATRA | n | -- | -- | y | -- | -- | -- | -- | -- | + |
| Olsson 1985 [32] | y | y | y | NR | ATRA | n | y | -- | y | -- | -- | -- | -- | Effects on morphology and expression of tumor associated antibodies | + |
| Wilkoff 1980 [33] | y | -- | -- | -- | ATRA | n | -- | -- | y | -- | -- | -- | -- | -- | + |
| Nettesheim 1976 (Cancer Res) [34] | -- | y | -- | 120 | ATRA | n | y | -- | -- | -- | -- | -- | -- | -- | + |
| Nettesheim 1976 (IntJCancer) [35] | -- | y | -- | 75 | ATRA, 13CRA, all trans retinyl acetate | n | y | m ↑mortality in ATRA group only | -- | -- | -- | -- | -- | -- | m |
| Fu 2007 [36] | y | y | -- | NR | Bex | n | y | -- | y | -- | -- | -- | -- | ↓ p-ERK 1/2 and p-JNK 1/2 | + |
| Tooker 2007 [37] | y | y | -- | 40 | Bex | w/w/o GCB | y | -- | y | -- | -- | -- | ↑ | -- | + |
| Wang 2006 [38] | -- | y | -- | 65 | Bex | n | y | -- | -- | -- | -- | -- | -- | -- | + |
| Yen 2006 [39] | y | y | -- | 32 | Bex | n | -- | -- | -- | -- | y | -- | -- | -- | + |
| Pereira 2006 [40] | -- | y | -- | NR | Bex | n | y | -- | -- | -- | -- | -- | -- | -- | + |
| Dragnev 2005 [41] | y | -- | -- | -- | Bex | erlotinib | -- | -- | y | -- | -- | -- | ↑ | -- | + |
| Hermann 2005 [42] | y | y | -- | 64-80 | Bex | PAX, VIN, CDDP,carboplatin | y | -- | y | -- | -- | -- | ↑ for paclitaxel and vinorelbine, but not platinum based drugs | | + |
| Yen 2004 [43] | y | y | -- | 32-40 | Bex | Pax | y | -- | y | -- | -- | -- | prevention and reversal of MDR phenotype | | + |
| Fazely 1988 [44] | y | -- | -- | -- | retinyl palmitate and acetate, ATRA | n | -- | -- | -- | -- | y | -- | -- | no direct cytotoxicity | + |
| Morrison 1981 [45] | -- | y | -- | 60 | retinyl palmitate | w/w/o vitC | y | -- | -- | -- | -- | -- | -- | -- | + |
| Pavelic 1980 [46] | -- | y | -- | 41 | retinyl palmitate | n | -- | n | -- | -- | -- | -- | -- | -- | n |
| Smith 1972 [47] | -- | y | -- | 159 | retinyl palmitate | n | n | -- | -- | -- | -- | -- | -- | -- | n |
| Saffiotti 1967 [48] | -- | y | -- | 144 | retinyl palmitate | n | y | n | -- | -- | -- | -- | -- | -- | + |
| Khanduja 1994 [49] | -- | y | -- | 140 | retinyl acetate | n | n | -- | -- | -- | -- | -- | -- | -- | n |
| Nettesheim 1975 [50] | -- | y | -- | NR | retinyl acetate | n | y | ↓mortality | -- | -- | -- | -- | -- | -- | + |
| Zhao 2005 [Abstr] [51] | y | -- | -- | -- | retinoic acid | n | -- | -- | y | -- | y | -- | -- | -- | + |
| Zhang 2003 [Abstr] [52] | y | -- | -- | -- | retinoic acid | n | -- | -- | y | -- | y | -- | -- | -- | + |
| Gaetano 1994 [53] | y | y | -- | 24 | retinoic acid | n | -- | -- | n | -- | y | -- | -- | Cell morphology: abolished malignant phenotype | + |
| Terasaki 1987 [54] | y | -- | -- | -- | retinoic acid | n | -- | -- | -- | -- | -- | -- | -- | Cell morphology: prevents and reverses carcinogenic changes in SCLC cells | + |
| Arbaje 1993 [55] | y | -- | -- | -- | cis and trans RA | w/w/o 5-FU, IFN | -- | -- | n | -- | -- | -- | no synergy | -- | n |
| Al-Wadei 2006 [56] | y | -- | -- | -- | 13CRA, 9CRA | n | -- | -- | m | -- | -- | -- | -- | ↑proliferation in small airway epithelial cells (eg, adenocarcinoma) | m |
| Frasca 1981 [57] | -- | y | -- | 250 | 13CRA, 9CRA | n | n | -- | -- | -- | -- | -- | -- | -- | n |
| Mernitz 2007 [58] | -- | y | -- | 126 | 9CRA | w/w/o 1,25  vitD | y | -- | -- | -- | -- | -- | -- | vitA ↓ toxicity of vitD | + |
| Mernitz 2006 [59] | -- | y | -- | 56-60 | 9CRA | celecoxib | y | ↑mortality | -- | -- | -- | -- | -- | ↑mortality in vitA +celecoxib groups vs 9CRA only; no celecoxib only group | m |
| Rosati 1998 [60] | y | -- | -- | -- | 9CRA, and 2 synthetic retinoids | n | -- | -- | y | y | -- | -- | -- | -- | + |
| Sun 1999 [61] | y | -- | -- | -- | 26 novel retinoids incl ATRA, 9CRA, 13CRA, 4-HPR | n | -- | -- | y | -- | -- | -- | -- | -- | + |
| Liby 2007 [62] | y | y | -- | -- | retinoid 4204 | n | y | -- | -- | y | -- | -- | -- | -- | + |
| Mourad 1996 [63] | y | -- | y | 54 | water soluble vitA (Aquasol) | n | -- | -- | n | -- | n | -- | -- | no effect on differentiation | n |
| Khanduja 1992 [64] | -- | y | -- | 140 | “vitA” ‡ | n | y | -- | -- | -- | -- | -- | -- | -- | + |
| Cattan 1986 [65] | -- | y | -- | -- | “vitA” ‡ | w/w/o etoposide, teniposide, CDDP | -- | no effect (↓ w CP) | -- | -- | -- | -- | none (-CP) | -- | n |
| Weinzweig 2003 [66] | -- | y | -- | 60 | “vitA” ‡ | n | -- | ↑ survival | -- | -- | n | -- | -- | no effect on mets to lung but increased survival | m |
| Kim 1979 [67] | -- | y | -- | NR | “vitA” ‡ | n | -- | -- | variable d/o cell line | -- | variable d/o cell line | -- | -- | -- | n |
| **TOTAL** | **49** | **28** | **2** | **--** | **--** | **--** | **14** | **3** | **33** | **15** | **7** | **2** | **--** | **cytotoxicity 3** | **+54** |

**References**

1. Kadara H, Tahara E, Kim HJ, Lotan D, Myers J, et al. (2008) Involvement of Rac in fenretinide-induced apoptosis. Cancer Res 68: 4416-4423.

2. Schroeder CP, Kadara H, Lotan D, Woo JK, Lee HY, et al. (2006) Involvement of mitochondrial and Akt signaling pathways in augmented apoptosis induced by a combination of low doses of celecoxib and 4HPR at clinically relevant concentrations. Cancer Res 66: 9762-9770.

3. Sun SY, Schroeder CP, Yue P, Lotan D, Hong WK, et al. (2005) Enhanced growth inhibition and apoptosis induction in NSCLC cell lines by combination of celcoxib and 4HPR at clinically relevant concentrations. Cancer Biology and Therapy 4: 407-413.

4. Ohlmann CH, Jung C, Jaques G (2002) Is growth inhibition and induction of apoptosis in lung cancer cell lines by fenretinide [N-(4-hydroxyphenyl)retinamide] sufficient for cancer therapy? Int J Cancer 100: 520-526.

5. Sun SY, Yue P, Kelloff GJ, Steele VE, Lippman SM, et al. (2001) Identification of retinamides that are more potent than N-(4-hydroxyphenyl)retinamde in inhibiting growth and inducing apoptosis of human head and neck and lung cancer cells. Cancer Epidemiol Biomarkers Prev 10: 595-601.

6. Maurer BJ, Melton L, Billups C, Cabot MC, Reynolds CP (2000) Synergistic cytotoxicity in solid tumor cell lines between N-94-hydroxyphenyl)retinamide and modulators of cermide metabolism. J Natl Cancer Inst 92: 1897-1909.

7. Kalemkerian GP, Ou X (1999) Activity of fenretinide plus chemotherapeutic agents in small-cell lung cancer cell lines. Cancer Chemotherapy and Pharmacology 43: 145-150.

8. Zou CP, Kurie JM, Lotan D, Zou CC, Hong WK, et al. (1998) Higher potency of N-(4-hydroxyphenyl)retinamide than all-transretinoic acid in induction of apoptosis in non-small cell lung cancer cell lines. Clinical Cancer Research 4: 1345-1355.

9. Kalemkerain GP, Slusher R, Ramalingam S, Gadgeel S, Mabry M (1995) Growth inhibition and induction of apoptosis by fenretinide in small-cell lung cancer cell lines. J Natl Cancer Inst 87: 1647-1680.

10. Choi EJ, Whang YM, Kim SJ, Kim HJ, Kim YH (2007) Combinational treatment with retinoic acid derivatives in non-small cell lung carcinoma in vitro. J Korean Med Sci 22 Suppl: S52-60.

11. Kadara H, Schroeder CP, Lotan D, Pisano C, Lotan R (2006) Induction of GDF-15/NAG-1/MIC-1 in human lung carcinoma cells by retinoid-related molecules and assessment of its role in apoptosis. Cancer Biology and Therapy 5: 518-522.

12. Kawakami S, Suzuki S, Yamashita F, Hashida M (2006) Induction of apoptosis in A549 human lung cancer cells by all-trans retinoic acid incorporated in DOTAP/cholesterol liposomes. J Control Release 110: 514-521.

13. Tabata C. Kubo, H, Tabata R, Wada M, Sakuma K, et al. (2006) All-trans retinoic acid modulates radiation-induced proliferation of lung fibroblasts via IL-6/IL-6R system. Am J Physiol Lung Cell Mol Physiol 290: L597-606.

14. Suzuki S, Kawakami S, chansri N, Yamashita F, Hashida M (2006) Inhibition of pulmonary metastasis in mice by all-trans retinoic acid incorporated in cationic liposomes. J Control Release 116: 58-63.

15. Lin LM, Li BX, Xiao JB, Lin DH, Yang BF (2005) synergistic effect of all-trans-retinoic acid an arsenic trioxide on growth inhibition and apoptosis in human hepatoma, breast cancer, and lung cancer cells in vitro. World J Gastroenterol 11: 5633-5637.

16. Ma Y, Feng Q, Sekula D, Diehl JA, Freemantle SJ, et al (2005) Retinoid targeting of different D-type cyclins through distinct chemopreventive mechanisms. Cancer Res 65: 6476-6483.

17. Maeno T, Tanaka T, Sando Y, Suga T, Maeno Y, et al (2002) Stimulation of vascular endothelial growth factor gene transcription by all trans retinoic acid through Sp1 and Sp3 sites in human bronchioloalveolar carcinoma cells. Am J Respir Cell Mol Biol 26: 246-253.

18. Albright CD, Grimley PM, Jones RT, Resau JH (2002) Differential effects of TPA and retinoic acid on cell-cell communication in human bronchial epithelial cells. Exp Mol Pathol 72: 62-67.

19. Fiorentini C, Facchetti M, Finardi A, Sigala S, Paez-Pereda M, et al. (2002) Nerve growth factor and retinoic acid interactions in the control of small cell lung cancer proliferation. Eur J Endocrinol 147: 371-379.

20. Hsu SL, Hsu JW, Liu MC, Chen LY, Chang CD (2000) Retinoic acid-mediated G1 arrest is associated with induction of p27(kip1) and inhibition of cyclin-dependent kinase 3 in human lung squamous carcinoma CH27 cells. Exp Cell Res 258: 322-331.

21. Manna SK, Aggarwal BB (2000) All-trans-retinoic acid upregulated TNF receptors and potentiates TNF-induced activation of nuclear factors-kappaB, activated protein-1 and apoptosis in human lung cancer cells. Oncogene 19: 2110-2119.

22. Agadir A, Chen G, Bost F, Li Y, Mercola D, et al. (1999) Differential effect of retinoic acid on growth regulation by phrbol ester inhuman cancer cell lines. J Biol Chem 274: 29779-29785.

23. Lokshin A, Zhang H, Mayotte J, Lokshin M, Levitt ML (1999) Early effects of retinoic acid on proliferation, differentiation and apoptosis in non-small cell lung cancer cell lines. Anticancer Res 19: 5251-5254.

24. Weber E, Ravi RK, Knudsen Es, Williams JR, Dillehay LE, et al. (1999) Retinoic acid-mediated growth inhibition of small cell lung cancer cells is associated with reduced myc and increased p27Kip1 expression. Int J cancer 80: 935-943.

25. Li Y, Dawson MI, Agadir A, Lee MO, Jong L, et al (1998) Regulation of RAR beta expression by RAR- and RXR- selective retinoids in human lung cancer cell lines: effect on growth inhibition and apoptosis induction. Int J Cancer 75: 88-95.

26. Liu G, Wu M, Levi G, Ferrari N (1998) Inhibition of cancer cell growth by all-trans retinoic acid and its analog N-(4-hydroxyphenyl)retinamide: a possible mechanism of action via regulation of retinoid receptors expresion. Int J Cancer 87: 248-254.

27. Guzey M, Demirpence E, Criss, W, DeLuca HF (1998) Effects of retinoic acid (all-trans and 9-cis) on tumor progression in small-cell lung carcinoma. Biochem Biophys Res Commun 242: 369-375.

28. Ross HJ (1996) The antiproliferative effect of trans-retinoic acid is associated with selective induction of interleukin-1 beta, a cytokine that directly inhibits growth of lung cancer cells. Oncol res 8: 171-178.

29. Higashimoto Y, Ohata M, Nishio K, Iwamoto Y, Fujimoto H, et al. (1996) 1 alpha, 25-dihydroxyvitamin D3 and all-trans-retinoic acid inhibit the growth of a lung cancer cell line. Anticancer Res 16: 2653-2659.

30. Kalemkerian GP, Jasti RK, Celano P, Nelkin BD, Mabry M (1994) All-trans-retinoic acid alters myc gene expression and inhibits in vitro progression in small cell lung cancer. Cell Growth Differ 5: 55-60.

31. Geradts J, Chen JY, Russell EK, Yankaskas JR, Nieves L, et al 91993) Human lung cancer cell lines exhibit resistance to retinoic acid treatment. Cell Growth Differ 4: 799-809.

32. Olsson L, Behnke O, Sorensen HR (1985) Modulatory effects of 5-azacytidine, phorbol ester, and retinoic acid on the malignant phenotype of human lung cancer cells. Int J Cancer 35: 189-98.

33. Wilkoff LJ, Dulmadge EA, Chopra DP (1980) Viability of cultured Lewis lung cell populations exposed to beta retinoic acid. Proc Soc Exp Biol Med 163: 233-236.

34. Nettesheim P, Cone MV, Snyder C (1976) The influence of retinyl acetate on the postinitiation phase of preneoplastic lung nodules in rats. Cancer Res 36: 966-1002.

35. Nettesheim P, Williams ML (1976) The influence of vitamin A on the susceptibility of the rat lung to 3-methylcholanthrene. Int J Cancer 17: 351-357.

36. Fu J, Ding Y, Huang D, Li H, Chen X (2007) The retinoid X receptor-selective ligand, LGD1069, inhibits tumor-induced angiogenesis via suppression of VEGF in human non-small cell lung cancer. Cancer lett 248: 153-163.

37. Tooker P, Yen WC, Ng SC, Negro-Vilar A, Hermann TW (2007) Bexarotene (LGD1069, Targretin), a selective retinoid X receptor agonist, prevents and reverses gemcitabine resistance in NSCLC cells by modulating gene amplification. Cancer Res 67: 4425-4433.

38. Wang Y, Zhang Z, Yao R, Jia D, Want D, et al. (2006) Prevention of lung cancer progression by bexarotene in mouse models. Oncogene 25: 1320-1329.

39. Yen WC, Prudente RY, Corpuz MR, Negro-Vilar A, Lamph WW (2006) A selective retinoid X receptor agonist bexarotene (LD1069, targretin) inhibits angiogenesis and metastasis in solid tumours. Br J Cancer 94: 654-660.

40. Pereira MA, Kramer PM, Nines R, Liu Y, Alyaqoub FS, et al. (2006) Prevention of mouse lung tumors by targretin. Int J Cancer 118: 2359-2362.

41. Dragnev KH, Petty WJ, Shah S, Biddle A, Desai NB, et al. (2005) Bexarotene and erlotinib for aerodigestive tract cancer. J Clin Oncol 23: 8757-8764.

42. Hermann TW, Yen WC, Tooker P, Fan B, Roegner K, et al. (2005) The retinoid X receptor agonist bexarotene (Targretin) synergistically enhances the growth inhibitory activity of cytotoxic drugs in non-small cell ung cancer cells. Lung Cancer 50: 9-18.

43. Yen WC, Corpuz MR, Prudente RY, Cooke TA, Bissonnette RP, et al (2004) A selective retinoid X receptor agonist bexarotene (Targretin) prevents and overcomes acquired paclitaxel (Taxol) resistance in human non-small cell lung cancer. Clinical Cancer Research 10: 8656-8664.

44. Fazely F, Ledinko N, Smith DJ (1988) Inhibition by retinoids of in vitro invasive ability of human lung carcinoma cells. Anticancer Res 8: 1387-1391.

45. Morrison DG, Daniel J, Lynd FT, Moyer MP, Esparza RJ, et al. (1981) Retinyl palmitate and ascorbic acid inhibit pulmonary neoplasms in mice exposed to fiberglass dust. Nutr Cancer 3: 81-85.

46. Pavelic ZP, Dave S, Bialkowski S, Priore RL, Greco WR (1980) Antitumor activity of Corynebacterium parvum and retinyl palmitate used in combination on the Lewis lung carcinoma. Cancer Res 40: 4617-4621.

47. Smith WE, Yazdi E, Miller L (1972) Carcinogenesis in pulmonary epithelia in mice on different levels of vitamin A Environ Res 5: 152-163.

48. Saffiotti U, Montesano R, Sellakumar AR, Borg SA (1967) Experimental cancer of the lung. Inhibition by vitamin A of the induction of tracheobronchial squamous metaplasia and squamous cell tumors. Cancer 20: 857-864.

49. Khanduja KL, Koul IB, Sehgal S (1994) Influence of combined deficiency of fat and vitamin A on benzo(a)pyrene-induced lung carcinogenesis in rats. Indian J Exp Biol 32: 124-127.

50. Nettesheim P. Snyder C, Williams ML (1975) Effect of vitamin A on lung tumor induction in rats. Proc Am Ass Cancer Res 16: 216.

51. Zhao Y, Li G, Yu R (2005) Experimental study of efefcts of retinoic acid on IL-1 beta and IFN-gamma induced C3 and factor B secretion in lung cancer cell line. Chinese Journal of Lung Cancer 8: 170-174.

52. Zhang D, Huang W, Huang J, Liao Z (2003) Studies on proliferation inhibition and anti-invasion of retinoic acid and 18beta-glycyrrhetinic acid in highly metastasized human lung cancer cell line. Chinese Journal of Lung Cancer 6: 181-184.

53. Gaetano C, Melchiori A, Albini A, Benelli R, Falcioni R, et al (1994) Retinoic acid negatively regulated beta 4 intregrin expression and suppresses the malignant phenotype in a Lewis lung carcinoma cell line. Clin Exp Metastasis 12: 63-72.

54. Terasaki T, Shimosato Y, Nakajima T, Tsumuraya M, Ichinose H, et al (1987) Reversible squamous cell characteristics induced by vitamin A deficiency in a small cell lung cancer cell line. Cancer Res 47: 3533-3537.

55. Arbaje TM, Bittner G, Yingling JM, Storer B, Schiller JH (1993) Antiproliferative effects of interferons –alpha and –beta in combination with 5-fluorouracil, cisplatin, and cis- and trans-retinoic acid in three human lung carcinoma cell lines. J Interferon Res 13: 25-32.

56. Al-Wadei HA, Schuller HM (2006) Cyclic adenosine monophosphate-dependent cell type-specific modulation of mitogenic signaling by retinoids in normal and neoplastic lung cells. Cancer Detect Prev 30: 403-411.

57. Frasca JM, Garfinkel L (1981) 13-cis retinoic acid and murine pulmonary adenomas: a preliminary report. Nutr Cancer 3: 72-74.

58. Mernitz H, Smith DE, Wood RJ, Russell RM, Wang XD (2007) Inhibition of lung carcinogenesis by 1 alpha, 25-dihydroxyvitamin D3 and 9-cis retinoic acid in the A/J mouse model: evidence of retinoid mitigation of vitamin D toxicity. Int J Cancer 120: 1402-1409.

59. Mernitz H, Smith DE, Zhu AX, Want XD (2006) 9-cis retinoic acid inhibition of lung carcinogenesis in the A/J mouse model is accompanied by increased expression of RAR-beta but no change in cyclooxygenase-2. Cancer Lett 244: 101-108.

60. Rosati R, Ramnath N, Adil MR, Ou X, Ali MA, et al (1998) Activity of 9-cis-retinoic acid and receptor-selective retinoids in small cell lung cancer cell lines. Anticancer Res 18: 4071-4075.

61. Sun SY, Kurie JM, You P, Dawson MI, Shroot B, et al (1999) Differential responses of normal, premalignant, and malignant human bronchial epithelial cells to receptro-selective retinoids. Clinical Cancer Research 5: 431-437.

62. Liby K, Royce DB, Risingsong R, Williams CR, Wood MD, et al. (2007) A new rexinoid, NRX194204, prevents carcinogenesis in both the lung and mammary gland. Clinical Cancer Research 13: 6237-6243.

63. Mourad WA, Bruner JM, Vallieres E, McName C, Alabdulwahed S, et al. (1996) The effect of high dose vitamin A on the morphology and proliferative activity of xenograft lung and head and neck cancer. In Vivo: 329-333.

64. Khanduja KL, Koul IB, Gandhi RK, Sehgal S, Sharma RR (1992) Effectof combined deficiency of fat and vitamin A on N-nitrosodiethylamine-induced lung carcinogenesis in mice. Cancer Lett 62: 57-62.

65. Cattan A, Bresson ML (1986) Vitamin A: failure to demonstrate in mice an anti-tumor effect, whether combined with cytotoxic drugs or not. Bull Cancer 73: 201-206.

66. Weinzweig J, Tattini C, Lynch S, Zienowicz R, Weinzweig N, et al. (2003) Investigation of the growth and metastasis of malignant melanoma in a murine model: the role of supplemental vitamin A. Plast Reconstr Surg 112: 152-158.

67. Kim JCS (1979) The effect of vitamin A on NO2 induced lung injury in hamsters. Journal of Applied Nutrition 31: 7-21.
