# Supplementary material for: Vitamin A and Retinoid Derivatives for Lung Cancer: A Systematic Review and Meta Analysis
Source: PLoS One. 2011 Jun 27;6(6):e21107. doi: 10.1371/journal.pone.0021107 (PMC3124481; doi:10.1371/journal.pone.0021107)
Supplement: Table S3 — Uncontrolled Phase I/II Trials for the Treatment of Lung Cancer in Order of the Form of Vitamin A. 5-FU 5-fluorouracil; adv advanced; bw between; CK: creatinine phosphokinase; CRA cis retinoic acid; d day; DLTs dose limiting toxicities; EFS event free survival; Gr Grade; IFN interferon; LFT's liver function tests; MIU = million IU; mo month; NS = non significant; NSCLC non small cell lung carcinoma; OS overall survival; PFS progression free survival; PHA phytohemagglutinin; pt patients; resp respectively; sign significant; SC subcutaneous; SCC small cell carcinoma; TC total cholesterol; TG triglyceride; TTP time to progression; w with; VEGF vascular endothelial growth factor; wk week; yr year. (DOC) [file pone.0021107.s003.doc]

**Table S3.** **Uncontrolled Phase I/ II Trials for the Treatment of Lung Cancer in Order of the Form of Vitamin A**

| **Reference** | **Trial Design and Characteristics** | **Form & Dose** | **Chemo or Additional Interventions** | **+/-/n** | **Outcomes** |
| --- | --- | --- | --- | --- | --- |
| **Positive Trials** | | | | | |
| Nandan 2006 [1] | Uncontrolled Phase II; n= 7 pt w adv NSCLC; retinoids given during first 4 days of chemo courses | tretinoin (ATRA) 100 mg/d + bexarotene 375 mg/d | docetaxel, capecitabine | + | **Efficacy:** 5 of 7 pts (71%) had an objective response; survival time ranged from 3-19 or more mo (>17 mo in 3 of 5 pt). |
|  |  |  |  |  | **Toxicity:** Not reported. |
|  |  |  |  |  | **Authors’ Interpretation:**  Based on the “high response rate” observed, “a formal phase II trial is recommended.” |
| Norsa 2006 [2] | Uncontrolled Phase II; n= 28 w adv NSCLC, no previous sx or chemo | ATRA 5mg, retinol 5000 IU | somatostatin, melato-nin, vitamin D, bromo-criptine, and cyclo-phosphamide, ßcarotene | + | **Efficacy:** Median survival 12.9 mo; 1 yr survival 51.2% (31.0-68.2); 2 yr survival 21.1% (7.0-40.2); improved symptoms of chest pain, cough, dyspnea, hemoptysis, fatigue, insomnia. |
|  |  |  |  |  | **Toxicity:** Gr 1/ 2 nausea, vomitting, diarrhea, drowsiness. |
|  |  |  |  |  | **Authors’ Interpretation:**  “Low performance status patients may benefit from [the combination] in terms of survival and quality of life.” |
| Thiruven-gadam 1996 [3] | Uncontrolled Phase II; n= 20 w adv NSCLC, chemo-naïve | ATRA 75 mg bid | cisplatin, etoposide | + | **Efficacy:** 53% pts had a partial response; median survival 25.5 wk; TTP was 23.5 wk (11-38 wk range); median time to partial response was 8 wk or 2 courses of tx; duration of response was 15.5 wk. |
|  |  |  |  |  | **Toxicity:** Gr 3/ 4 neutropenia, thrombocytopenia. Gr 1 mucocutaneous symptoms (dryness, epistaxis), reversible hypercalcemia (2), reversible nephrotoxicity (5). |
|  |  |  |  |  | **Authors’ Interpretation:**  This regimen has” significant activity in advanced NSCLC.” |
| Athanasi-adis 1995 [4] | Uncontrolled Phase II; n= 29 w adv NSCLC | ATRA 150 mg/m^2^ | IFN-α 3 MIU SC | + | **Efficacy:** Objective responses seen in 16% of patients; median survival was 8 mo, with significant difference in survival b/w responders and non-responders (p=0.008). |
|  |  |  |  |  | **Toxicity:** ↑TG in 44% of patients; Gr 1 mucocutaneous symptoms, headache, fever, fatigue. No hematological toxicity. |
|  |  |  |  |  | **Authors’ Interpretation:**  Combined differentiation treatment has “modest but objective activity in NSCLC.” |
| Recchia 2006 [5,6] | Non-randomized, controlled Phase II trial; n= 38 pt with adv NSCLC matched to 87 controls who had similar responses to the same chemotherapy | 13 CRA 0.5 mg/kg | IL-2 1.8 x10^6^ IU SC | + | **Efficacy:** Sign ↑ survival time by 11% at 2 yr with median survival difference of 11.6 mo. overall; median PFS (16.5 vs 8.4 mo). No sign difference in response rates. ↑NK, ↑CD4/CD8 ratio, and ↓VEGF levels. |
|  |  |  |  |  | **Toxicity**: Gr 1/ 2 fever, ↑TG, hematologic and cutaneous toxicities, and mild hypothyroidism seen in intervention group. |
|  |  |  |  |  | **Authors’ Interpretation:**  Administration of this combination “seems to improve progression free and overall survival.” |
| Recchia 2001 [7] | Uncontrolled Phase I; n= 18 w NSCLC (8) and other cancers | 13 CRA 0.5 mg/kg | IL-2 (dose ranging) | + | **Efficacy:** Overall response rate: 54.4% (95% CI: 43.5–64.9%). 4 patients (22.2%) were alive, and 3 of them (16.6%) disease-free at 25 mo. Median TTP was 8.1 months. Median survival was 13.7 mo., and 1-and 2-year survival rates were 62 and 28%, respectively. ↑ in immunological parameters was also seen. |
|  |  |  |  |  | **Toxicity:** No patients at dose level three had any Gr3 or 4 toxicity. Optimal biological dose of IL-2 and RA were 1.8 MIU and 0.5 mg/kg body weight respectively. |
|  |  |  |  |  | **Authors’ Interpretation:**  This combination as maintenance therapy after chemotherapy is “feasible and well tolerated and improves immunological parameters known to have a prognostic value in cancer.” |
| Recchia 2000 [8] | Uncontrolled Phase II; n= 30 w adv NSCLS | 13 CRA 1.0 mg/kg bw chemo courses | cisplatin, vindesine, mitomycin-C | + | **Efficacy:** 40% pts had an objective response, and 12 pt had objective remission; TTP 9 mo (range 3.9-45+ mo); 1 yr survival was 47% |
|  |  |  |  |  | **Toxicity:** Gr 2 diarrhea (20%), transient ileus (10%), febrile neutropenia, ↑LFTs, hyperlipidemia; Gr 3/ 4 hematological toxicity (16%), nausea and vomitting, anemia, alopecia. |
|  |  |  |  |  | **Authors’ Interpretation:**  The addition of RA to this chemotherapy is “feasible and shows some acitivty in the treatment of NSCLC, with manageable toxicity.” |
| Recchia 1999 [9] | Uncontrolled Phase II; n= 28 w adv NSCLC (stage IIIB or IV) | 13 CRA 1 mg/kg | carboplatin, vindesine, leucovorin, 5-FU | + | **Efficacy:** Overall response rate was 39% (95% CI 22-60%). Median survival was 9.7 months (range 0.5-27 mo.). Median TTP was 7.7 months (range 3.4-22 mo). |
|  |  |  |  |  | **Toxicity:** ↑TG (2x baseline values) in 11% of patients. Other toxicities may have been related to chemotherapy: Gr 3-4 hematological toxicity in 46%, Gr2 diarrhea in 21%, ileus in 14%, and Gr2 neurological symptoms in 11%. |
|  |  |  |  |  | **Authors’ Interpretation:** The addition of retinoic acid to this chemotherapy regimen “represents an effective treatment in NSCLC, with managable toxicity.” |
| Dragnev 2005 [10] | Uncontrolled Phase I; n=24 w advanced aerodigestive cancers, of which 19 (79%) were NSCLC | bexarotene 200, 300, 400 mg/m^2^ | erlotinib 100 or 150 mg | + | **Efficacy:** 9 of 24 patients had disease stabilization lasting 8 to 70+ wks. Survival at one yr was 72% among NSCLC patients, and median TTP was 2.0 mo. This was reportedly better than results found previously with erlotinib alone. The MTD was not reached. |
|  |  |  |  |  | **Toxicity:** most common were mucocutaneous symptoms, mild ↑TG, hypothyroidism. **DLT**s: one case of a pain syndrome deemed treatment related, and one case of ↑CK deemed secondary to antilipid therapy. |
|  |  |  |  |  | **Authors’ Interpretation:**  “Overall survival and clinical features of responding patients differ from prior reports of single-agent erlotinib treatment.” |
| Edelman 2005 [11] | Non-randomized, controlled Phase II; n= 48 w adv NSCLC; Bexarotene plus chemo vs historical controls | bexarotene 400 mg/m^2^ | carboplatin, gemcitabine | + | **Efficacy:** Sign ↑ median OS 12.7 vs 9.3 mo and median TTP 6.7 vs 3.9 mos in treatment group vs controls resp; median EFS was 7.3 mo in pt w St IIIB cancer, and 6.5 mo in total group. 1 yr survival 53% and response rate 25% in bexarotene group. |
|  |  |  |  |  | **Toxicity**: ↑TG requiring statins up to 40 mg/d; hypothyroidism (2) |
|  |  |  |  |  | **Authors’ Interpretation:**  “The median time to progression and overall survival are promising and warrant further evaluation.” |
| Khuri 2001 [12] | Uncontrolled Phase I n=21; Uncontrolled Phase II n=28. Pt w Stage IIIB or IV NSCLC, chemo-naïve | bexarotene dose-escalation 0-600 mg/m^2^ | vinorelbine, cisplatin | + | **Efficacy:** MTD in combination with cisplatin + vinorelbine = 400 mg/m^2^. Overall response rate 25%; 50% had disease stabilization. Median survival 593 d (range, 273-1284+ days). One year survival rate 61%; projected 3-yr survival rate was 30%, which were deemed remarkable findings compared to typical Phase II findings. |
|  |  |  |  |  | **Toxicity:** most common were asthenia (81%), leukopenia (74%), nausea (70%), hyperlipidemia (67%), vomiting (58%), headache (56%), dermatitis (56%), and anorexia (51%). |
|  |  |  |  |  | **Authors’ Interpretation:**  Treatment led to “acceptable phase II response rates and was associated with better-than-expected survival.” |
| Recchia 1997 [13] | Uncontrolled Phase II; n= 40 w adv NSCLC, chemo-naive | retinyl palmitate 50,000 IU bid | IFN-ß, 5-FU, cisplatin, vindesine | + | **Efficacy:** 42.5% pts had an objective response (3 complete, 14 partial, 14 had stable disease, and 9 had progressive disease). Median survival was 9.1 mo, median response duration 5.1 mo (range 2.5-104 mo), and survival at 1 yr was 35%. |
|  |  |  |  |  | **Toxicity:** Gr 2 ↑TG, mucocutaneous symptoms, hematological toxicity in 30%. Gr 3/ 4 diarrhea, alopecia, leukopenia. Mild elevation of LFTs in 3 patients. |
|  |  |  |  |  | **Authors’ Interpretation:**  This combination “shows activity in NSCLC, particularly in tumors with squamous origin.” |
| Micksche 1977 [14] | Uncontrolled Phase II; n= 9 pt w non resectable SCC (lung) | retinyl palmitate 1.5 MIU/d or 13 CRA 100 mg/d | none | + | **Efficacy:** After treatment with both forms of VitA, sign incr (p<0.001) in reactivity against the antigen PHA was detected *in vitro*, compared to pre-treatment values. |
|  |  |  |  |  | **Toxicity:** generally mild, mucocutaneous symptoms most common. |
|  |  |  |  |  | **Authors’ Interpretation:**  Results support the immune potentiating properties of vitamin A in lung cancer patients. |
| **Trials Showing No Significant Effect** | | | | | |
| Govindan 2006 [15] | Uncontrolled Phase II single arm trial; n=146 w Stage IIIB or IV NSCLC after failure of two or more previous therapies. | bexarotene 400 mg/m^2^ | Antilipid therapy + levothyroxine prophylactically | n | **Efficacy:** By intention to treat analysis, the study did not achieve the endpoint of 6 mo median survival. Of the patients who received bexarotene for ≥2 wk, median survival was 6 mo (5-8 mo). Patients with bexarotene induced hypertriglyceridemia had sign longer median survival compared to those with normal triglyceride (p=0.004). |
|  |  |  |  |  | **Toxicity:** most common ↑TG (6), nausea & vomiting (5), skin rash (2), low blood counts (2), confusion (2). |
|  |  |  |  |  | **Authors’ Interpretation:**  “Bexarotene given as third or subsequent line of therapy for relapsed NSCLC did not achieve the intended median survival of 6 months.” |
| Wildi 2008 [16] | Uncontrolled Phase I; n=15 w solid tumors (w no curative options), 10 w NSCLC | bexarotene 200, 300, 400 mg/m^2^ | docetaxel, dexa-methasone, atrovastatin; thyroxine as needed. | n | **Efficacy:** 4 patients had disease stabilization; of these, 3 with NSCLC stabilized for a mean of 24.1 wk, and all 4 had grade 2-3 ↑TG. The MTD was not reached. |
|  |  |  |  |  | **Toxicity:** neutropenia was the most common hematologic toxicity. Most common non hematological toxicities were ↑TG and hypothyroidism. |
|  |  |  |  |  | **Authors’ Interpretation:**  Treatment was well tolerated. “Given that stable disease was durable in 4 patients, future studies with [this combination] may be warranted.” |
| Rizvi 1999 [17] | Uncontrolled Phase I; n=60 w advanced cancer, 16 with lung cancer | bexarotene dose escalation, micronized form |  | n | **Efficacy:** No objective tumor responses seen, but agent may have contributed to disease stabilization in 5 of the 16 NSCLC patients. Recommended Phase II dose 500 mg/m^2^. |
|  |  |  |  |  | **Toxicity**: thyroid function: At dosages above 200 mg/m^2^, there was a dose-dependent ↓ in both TSH and T4, and these normalized on discontinuation of treatment. There were no clinical symptoms of hypothyroidism except fatigue. |
|  |  |  |  |  | **Authors’ Interpretation:**  Although there was no objective response, the findings “support further study” of this agent. |
| Meyskens 1981 [Abstr] [18] | Uncontrolled Phase II; n= 134 w adv cancer | 13 CRA 3 mg/kg | none | n | **Efficacy:** 1 objective response among 11 lung cancer patients. |
|  |  |  |  |  | **Toxicity:** mucocutaneous symtpoms, emotional lability, depression. |
|  |  |  |  |  | **Authors’ Interpretation:**  “These early results are encouraging and suggest that RA has anticancer activity against epithelial malignancies.” |
| Grunberg 1987 [19] | Uncontrolled Phase II; n= 25 w adv NSCLC | 13 CRA 100 mg/m^2^ | none | n | **Efficacy:** 1 partial response. |
|  |  |  |  |  | **Toxicity:** mucocutaneous symptoms, ↑TG, headache. 1 patient had ↑transaminases. |
|  |  |  |  |  | **Authors’ Interpretation:**  “Activity of oral isotretinoin in advanced NSCLC appears to be minimal.” |
| Arnold 1994 [20] | Uncontrolled Phase II; n= 34 w NSCLC | 13 CRA 1 mg/kg | IFN-α 3 MIU SC | n | **Efficacy:** Response rates: 1 objective response, 23 had progressive disease, 8 had stable disease. Overall, no sign of effect was seen. |
|  |  |  |  |  | **Toxicity:** most common lethargy (32%), including 5 Gr3 cases. One Gr 4 toxicity: reversible ↑ LFTs and jaundice. |
|  |  |  |  |  | **Authors’ Interpretation:**  Results suggest that this combination is “virtually inactive in untreated patients with advanced NSCLC.” |
| Roth 1994 [21] | Uncontrolled Phase II; n=10 w SCC (lung) and head & neck cancers | 13 CRA 2 mg/kg | IFN-α 6 MIU SC | n | **Efficacy:** No objective responses seen. |
|  |  |  |  |  | **Toxicity:** Gr 1/2 mucocutaneous symptoms, fatigue, fever, anorexia. One Gr 3 toxicity: erosion of right lower eyelid and episcleritis in one patient. |
|  |  |  |  |  | **Authors’ Interpretation:**  Although there was no response to treatment, toxicity was surprisingly low and suggests that IFN may alleviate retinoid associated toxicity. |
| Rinaldi 1993 [22] | Uncontrolled Phase II; n= 21 w adv SCC (lung) | 13 CRA 1 mg/kg | IFN-α 3 MIU SC | n | **Efficacy:** No sign effects seen: 1 partial response, median survival of 31 weeks. |
|  |  |  |  |  | **Toxicity:** Gr 1/2 mucocutaneous symptoms, fever. Gr 3 fatigue, anorexia, nausea, headache in a total of 9 patients. |
|  |  |  |  |  | **Authors’ Interpretation:**  This combination is “unlikely to exhibit significant clincial activity in patients with metastatic SCC lung cancer.” |
| Kurie 1996 [23] | Uncontrolled Phase I; n= 22 w solid tumors including NSCLC | 9 CRA 20-150 mg/m^2^ | none | n | **Efficacy:** No objective responses. |
|  |  |  |  |  | **Toxicity:** Gr 1/2 mucocutaneous symptoms, gastrointestinal symptoms, ↑TG, ↑LFTs. Gr 3 ↑TG, ↑TC within 1 week of treatment. |
|  |  |  |  |  | **Authors’ Interpretation:**  No further interpretation provided wrt presumed efficacy. |
| Kalemke-rian 1998 [24] | Uncontrolled Phase II; n= 23 w adv SCLC | ATRA 150 mg/m^2^ | cisplatin, etoposide | n | **Efficacy:** 10 objective responses (45.5%); median duration of response was 5.1 mo, survival at one year was 40.9%, and median survival was 10.9 mo. |
|  |  |  |  |  | **Toxicity:** hematological toxicity (21 episodes of Gr4, 26 episodes of Gr3 toxicity), Gr 4 nausea, vomitting or diarrhea in 2 patients; Gr 2 ↑ ALP, ↑TG, ↑TC common. **DLTs** were mucocutaneous symptoms, headache, fatigeu, nausea & vomitting, myalgia, confusion. |
|  |  |  |  |  | **Authors’ Interpretation:**  Toxicity was significant, and “response rates and suvival were similar to those associatd with [these chemo drugs]in previous trials.” |
| Treat 1996 [25] | Uncontrolled Phase II; n= 28 w metastatic NSCLC | ATRA  175 mg/m^2^ | none | n | **Efficacy:** Median survival 7 mo; 2 partial responses, 20 pt had progressive disease, 2 had stable disease. |
|  |  |  |  |  | **Toxicity:** Gr 1/2 mucocutaneous symptoms in nearly all patients, headache (18), myalgia (9), nausea & vomiting (8). Gr 3 headache in 2 patients. ↑TG > 1.5 fold (24), ↑ AST/ ALT (15), ↑ WBC (3). |
|  |  |  |  |  | **Authors’ Interpretation:**  ATRA has “minimal activity as a single agent in NSCLC.” |
| Lee 1993 [26] | Uncontrolled Phase I; n= 40 w solid cancers including lung | ATRA 45-200 mg/m^2^ | none | n | **Efficacy:** No major objective responses in lung cancer pts; 150-175 mg/m^2^/d maximum tolerated dose (MTD) in adults. |
|  |  |  |  |  | **Toxicity:** Gr 1/2 mucocutaneous symptoms and headache most common. Gr 3 skin toxicity at 175 mg/m^2^ dosage or higher in 4 of 11 patients. |
|  |  |  |  |  | **Authors’ Interpretation:**  No further interpretation provided wrt presumed efficacy. |
| Goodman 1986 [27] | Uncontrolled Phase II; n= 65 w adv cancer, including NSCLC (17) | retinol 200,000 IU/m^2^ | none | n | **Efficacy:** 1 partial response, 5 mixed responses seen. |
|  |  |  |  |  | **Toxicity:** 62% had no seide effects. Mucocutaneous symptoms in 13%. Neurological symptoms in 20%. ↑TG to mean of 114.6 mg/dL (p<0.0005 vs baseline). |
|  |  |  |  |  | **Authors’ Interpretation:**  “Oral retinol appears to have limited activity in patients with advanced cancer.” |
| **Proof of Principle/ Biomarker Studies (1)** | | | | | |
| Dragnev 2007 [28] | Open label, uncontrolled single arm study; n=12 w primarily stage I-II NSCLC | Bexarotene 300 mg/m^2^ for 7-9 days prior to resection | | n | **Efficacy:** Two cases with the highest tumor tissue levels of bexarotene had changes in expression of several biomarkers including cyclin D3, EGFR, pEGFR, and Ki-67. Such changes were seen only if tumor tissue concentration was very high (>100 ng/g) and this occurred only in 3 cases. |
|  |  |  | |  | **Authors’ Interpretation:**  “Bexarotene represses proliferation and biomarker expression in responsive, but not resistant lung cancer cells.” |

**References**

1. Nandan R (2006) Promising results achieved with a combination of chemotherapy and two retinoids in patients with advanced non-small-cell lung cancer [1]. Lung Cancer 51: 387-388.

2. Norsa A, Martino V (2006) Somatostatin, retinoids, melatonin, vitamin D, bromocriptine, and cyclophosphamide in advanced non-small-cell lung cancer patients with low performance status. Cancer Biother Radiopharm 21: 68-73.

3. Thiruvengadam R, Atiba JO, Azawi SH (1996) A phase II trial of a differentiating agent (tRA) with cisplatin-VP 16 chemotherapy in advanced non-small cell lung cancer. Invest New Drugs 14: 395-401.

4. Athanasiadis I, Kies MS, Miller M, Ganzenko N, Joob A, et al. (1995) Phase II study of all-trans-retinoic acid and alpha-interferon in patients with advanced non-small cell lung cancer. Clin Cancer Res 1: 973-979.

5. Recchia FS, G. Nuzzo, A. (2006) Maintenance immunotherapy in lung cancer with low-dose interleukin-2 and 13-cis retinoic acid. Enhancer - Biotherapy of Cancer 4: 21-24.

6. Recchia F, Saggio G, Nuzzo A, Biondi E, Di Blasio A, et al. (2006) Multicenter phase 2 study of interleukin-2 and 13-cis retinoic acid as maintenance therapy in advanced non-small-cell lung cancer. J Immunother 29: 87-94.

7. Recchia F, De Filippis S., Rosselli M., Saggio G., Cesta A., Fumagalli L., Rea S. (2001) Phase 1B study of subcutaneously administered interleukin 2 in combination with 13-cis retinoic acid as maintenance therapy in advanced cancer. Clinical Cancer Research 7: 1251-1257.

8. Recchia F, Sica G, De Filippis S, Rosselli M, Saggio G, et al. (2000) Cisplatin, vindesine, mitomycin-C and 13-cis retinoic acid in the treatment of advanced non small cell lung cancer. A phase II pilot study. Anticancer Res 20: 1985-1990.

9. Recchia F, De Filippis S., Pompili P.L., Rosselli M., Saggio G., Ciorra A., Piccinini M., Rea S. (1999) Carboplatin, vindesine, 5-fluorouracil-leucovorin and 13-cis retinoic acid in the treatment of advanced non-small cell lung cancer. A phase II study. . Clinica Terapeutica 150: 269-274.

10. Dragnev KH, Petty WJ, Shah S, Biddle A, Desai NB, et al. (2005) Bexarotene and erlotinib for aerodigestive tract cancer. Journal of Clinical Oncology 23: 8757-8764.

11. Edelman MJ, Smith R, Hausner P, Doyle LA, Kalra K, et al. (2005) Phase II trial of the novel retinoid, bexarotene, and gemcitabine plus carboplatin in advanced non-small-cell lung cancer. Journal of Clinical Oncology 23: 5774-5778.

12. Khuri FR, Rigas JR, Figlin RA, Gralla RJ, Shin DM, et al. (2001) Multi-institutional phase I/II trial of oral bexarotene in combination with cisplatin and vinorelbine in previously untreated patients with advanced non-small-cell lung cancer. Journal of Clinical Oncology 19: 2626-2637.

13. Recchia F, Sica, G., De Filippis, S., Rea, S., Frati, L. (1997) Combined chemotherapy and differentiation therapy in the treatment of advanced non-small-cell lung cancer. . Anticancer Research 17: 3761-3765.

14. Micksche M, Cerni C, Kokron O, Titscher R, Wrba H (1977) Stimulation of immune response in lung cancer patients by vitamin A therapy. Oncology 34: 234-238.

15. Govindan R, Crowley J, Schwartzberg L, Kennedy P, Williams C, et al. (2006) Phase II trial of bexarotene capsules in patients with advanced non-small-cell lung cancer after failure of two or more previous therapies. Journal of Clinical Oncology 24: 4848-4854.

16. Wildi JD, Baggstrom MQ, Suresh R, Read W, Fracasso PM, et al. (2008) A phase I study of docetaxel and bexarotene. Chemotherapy 54: 125-130.

17. Rizvi NA, Marshall JL, Dahut W, Ness E, Truglia JA, et al. (1999) A phase I study of LGD1069 in adults with advanced cancer. Clinical Cancer Research 5: 1658-1664.

18. Meyskens JFL, Gilmartin E., Chase E. (1981) A broad phase II trial of 13-cis-retinoic acid in advanced cancer. . Proceedings of the American Association for Cancer Research 22: C-151.

19. Grunberg SM, Itri LM (1987) Phase II study of isotretinoin in the treatment of advanced non-small cell lung cancer. Cancer Treat Rep 71: 1097-1098.

20. Arnold A, Ayoub J, Douglas L, Hoogendoorn P, Skingley L, et al. (1994) Phase II trial of 13-cis-retinoic acid plus interferon alpha in non-small-cell lung cancer. The National Cancer Institute of Canada Clinical Trials Group. J Natl Cancer Inst 86: 306-309.

21. Roth AD, Abele R, Alberto P (1994) 13-cis-retinoic acid plus interferon-alpha: a phase II clinical study in squamous cell carcinoma of the lung and the head and neck. Oncology 51: 84-86.

22. Rinaldi DA, Lippman SM, Burris HA, 3rd, Chou C, Von Hoff DD, et al. (1993) Phase II study of 13-cis-retinoic acid and interferon-alpha 2a in patients with advanced squamous cell lung cancer. Anticancer Drugs 4: 33-36.

23. Kurie JM, Lee JS, Griffin T, Lippman SM, Drum P, et al. (1996) Phase I trial of 9-cis retinoic acid in adults with solid tumors. Clin Cancer Res 2: 287-293.

24. Kalemkerian GP, Jiroutek M, Ettinger DS, Dorighi JA, Johnson DH, et al. (1998) A phase II study of all-trans-retinoic acid plus cisplatin and etoposide in patients with extensive stage small cell lung carcinoma: an Eastern Cooperative Oncology Group Study. Cancer 83: 1102-1108.

25. Treat J, Friedland D, Luginbuhl W, Meehan L, Gorman G, et al. (1996) Phase II trial of all-trans retinoic acid in metastatic non-small cell lung cancer. Cancer Invest 14: 415-420.

26. Lee JS, Newman RA, Lippman SM, Huber MH, Minor T, et al. (1993) Phase I evaluation of all-trans-retinoic acid in adults with solid tumors. J Clin Oncol 11: 959-966.

27. Goodman GE (1986) Phase II trial of retinol in patients with advanced cancer. Cancer Treat Rep 70: 1023-1024.

28. Dragnev KH, Petty WJ, Shah SJ, Lewis LD, Black CC, et al. (2007) A proof-of-principle clinical trial of bexarotene in patients with non-small cell lung cancer. Clinical Cancer Research 13: 1794-1800.
