# Supplementary material for: Vitamin A and Retinoid Derivatives for Lung Cancer: A Systematic Review and Meta Analysis
Source: PLoS One. 2011 Jun 27;6(6):e21107. doi: 10.1371/journal.pone.0021107 (PMC3124481; doi:10.1371/journal.pone.0021107)
Supplement: Table S4 — Methodologies of Controlled Human Studies for Vitamin A and Lung Cancer. CA cancer; CAD coronary artery disease; comb combination; f/u follow up; GI gastrointestinal; LTFU loss to follow up; NR not reported; NSCLC non small cell lung cancer; pop population; PS performance score; SCLC small cell lung cancer; w with. (DOC) [file pone.0021107.s004.doc]

**Table S4. Methodologies of Controlled Human Studies for Vitamin A and Lung Cancer**

| **Ref.** | **Population** | | | | | | | **Intervention** | | **Control** | **Treat-ment duration** | **Sample size** | **Study duration** | **Blinding** | **Random** | **Drop-outs / LTFU reported** | **JADAD Score (RCTs)** |
| --- | --- | --- | --- | --- | --- | --- | --- | --- | --- | --- | --- | --- | --- | --- | --- | --- | --- |
|  | **Age** | **Gender** | **Smokers/ asbestos exposure** | **Staging** | **PS** | | **Previous chemo or surgery** |  | |  |  |  |  |  |  |  |  |
| **RCTs - Treatment** | | | | | | | | | | | | | | | | | |
| Recchia 2005 [1] | mean (range): T 53.1 (27-80) C 51 (21-81) | M 58; F 54 | NR | Stage III or IV: breast, lung (n=26), GI, or other CA | ECOG PS 0, 1, or 2 | | chemo | 13CRA (0.5 mg/kg/d) + IL-2 (1.8 MIU) | | IL-2 only | 1 y | 112 (lung 26) | 4 y (2000/2001 to Sept 2004) | n | y | n | **1** |
| Ramlau 2008 [2] | median 61 | M 449; F 174 | NR | Stage IIIB w pleural effusion or IV NSCLC | ECOG PS 0-1 | | NR | Bexarotene (400 mg/m2/d) +cisplatin/ vinorelbine | | cisplatin and vinorelbine only | 12-13 wk | 623 | NR: median follow up 24.5 mo | n | y | y | **2** |
| Blumenschein 2008 [3] | median 63 | M 132; F 480 | NR | Stage IIIB or IV NSCLC | ECOG PS 0-1 | | none | Bexarotene (400 mg/m2/d) + antilipid therapy + carboplain/ paclitaxel | | paclitaxel and carboplatin only | 11-13 wk | 612 | NR: median follow up 21.8 mo, but 2-yr survival was a secondary outcome | n | y | y | **2** |
| Ruotsalainen 2000 [4] | median (range): 61 (39-74) | M 58; F 27 | NR | Stage I-IV SCLC | KPS ≥ 60 | | chemo | Arm 1: 13CRA (1 mg/kg/d) + IFN α-2a (6 MIU 3x/wk x 4 wk, then 3 MIU 3x/wk); Arm 2: trophosphamide 100-150 mg/d | | Arm 3: control, no maintenance therapy | 1 y | 60 | NR: survival data is given up to 41 mo | n | y | n | **2** |
| Rizvi 2001 [5] | median 64 | M 26; F 26 | NR | NSCLC: Stage IIIB w pleural effusion, Stage IV, or recurrent w stable or responsive disease | ECOG PS 0, 1, or 2 | | chemo | Bexarotene (300 or 600 mg/m2/d) following firstline platinum based chemo | | same chemotherapy followed by placebo | until progression or withdrawal | 52 | 4 y (1996/8 to Jan 2000) | double | y | n | **4** |
| **Primary Prevention** | | | | | | | | | | | | | | | | | |
| Omenn 1996 (CARET) [6] | mean 57 +/-7 (asbestos); 58 +/-5 (smokers) | M 12,025, F 6289 | y | n/a | n/a | | n/a | Retinyl palmitate (25,000 IU/d) + 30 mg Beta carotene (30 mg/d) | | placebo | 5.5 y | 18314 | 12 y (1983/94 to Dec 1995) | double | y | n | **4** |
| de Klerk 1998 [7] | mean 57 | M 947, F 77 | y | n/a | n/a | | n/a | Retinyl palmitate (25,000 IU/d) | | beta carotene 30 mg/d | 400 d | 1024 | 5 y (June 1990 to May 1995) | single | y | n | **2** |
| Kamangar 2006 [8] | range 40-69 | M 13,313, F 16,271 | 30% smokers | n/a | n/a | | n/a | Retinyl palmitate (25,000 IU/d) + zinc (22.5 mg/d) in comb. w other arms (2(4) factorial design) | | placebo | 5.25 y | 28584 | 15 y (1986 to 2001) | double | y | n | **4** |
| **Secondary Prevention** | | | | | | | | | | | | | | | | | |
| Pastorino 1993 [9] | NR | NR | NR | Stage Ia resected NSCLC | n/a | | surgery | Retinyl palmitate (300,000 IU/d) | | no treatment | 1 y | 181 | 7 y (1985/9 to July 1992) | n | y | y | **2** |
| van Zandwijk 2000 [10] | median (range) 61 (19-91) | M 2238, F 335 | 93.5% current/ former smokers | H&N or NSCLC; NSCLC (40% of total n); staging pT1-2, N0-1, T3N0 | n/a | | surgery | Arm 1: Retinyl palmitate (300,000 IU/d x 1 yr, 150,000 IU/d for 2nd year); Arm 2: NAC (600 mg/d); Arm 3: retinyl palmitate + NAC | | no treatment | 2 y | 2573 (lung 40%) | up to 10y after randomization; total f/u of 8923 person-years | n | y | y | **3** |
| Lippman 2001 [11] | mean (SD) 64.2 (8.8) | M 667, F 499 | 39.1% current, 53.2% former smokers | Stage I NSCLC w resection currently ds free; TNM staging of T1-2 N0 M0 | n/a | | surgery | Isotretinoin (10-30 mg q2d) | | placebo | 3 y | 1166 | 7 y (final analysis at 7y) | double | y | n | **4** |
| **Observational – Prospective cohorts** | | | | **Ethnicity** | |  | | | **Measure of VitA** |  | **Cases** | **Cohort** |  |  | | | |
| Alfonso 2006 (Wittenoom cohort) [12] | median for each group 59, 61, and 51 | M 1399, W 554 | y | Australian | n/a | | n/a | serum retinol | | -- | 47 lung, 65 mesothelioma | 1953 | 12 y (1990-2002) | n | n | n | -- |
| Holick 2002 (ATBC cohort) [13] | range 50-69 | men only | y | Finnish | n/a | | n/a | serum retinol (+ dietary intake) | | -- | 1644 | 27084 | 14 y (1985-1998; incl. intervention period of ATBC study) | n | n | n | -- |
| Stahelin 1991 (Basel cohort) [14] | mean 48 +/- 10 | men only | 33.4% of controls and 72.1% of lung cancer cases were smokers | Swiss | n/a | | n/a | serum retinol | | -- | 68 | 2974 | 12 y (1971/3 to 1985) | n | n | y | -- |
| Ito 2005 (Hokkaido cohort) [15] | range 39-79 | M 1239, F 1943 | 26.8% current smokers | Hokkaido Japan residents | n/a | | n/a | serum retinol | | -- | 31 | 3182 | 10.5 y | n | n | y | -- |
| *Ito 2006 (Hokkaido cohort) [16]* | range 39-79 | M 1260, F 1994 | 25.4% current smokers | Hokkaido Japan residents | n/a | | n/a | serum retinol | | -- | 41 | 3254 | 11.7 y | n | n | y | -- |
| Yong 1997 (NHANES I cohort) [17] | median 50 (range 25-74) | M 3968, F 6100 | 31% current, 17% former smokers | American: 83% white, 16% black, 1% other | n/a | | n/a | supplemental vitamin A (+ dietary intake) | | -- | 248 | 10068 | 21 y (1971/5 to 1992) | n | n | y | -- |
| Paganini-Hill 1987 (Leisure world cohort) [18] | median 74 | two thirds women | Smokers and non (% not given) | California retirement home residents | n/a | | n/a | supplemental vitamin A (+ dietary intake) | | -- | 56 | 10473 | 5 y (1981-1986) | n | n | n | -- |
| Satia 2009 (VITAL cohort) [19] | mean 67.0 +/-6.8 (cases); 61.9 +/-7.4 (ctl) | M 37,053, F40,073 | Current smokers, n=6424 | Washington state residents: white 70,619, non-white 5175 | n/a | | n/a | supplemental vitamin A and beta carotene | | -- | 521 | 77126 | 5 y (2000/2 to 2005) | n | n | y | -- |
| **Observational – Case Control and Nested Case Control** | | | | | | | | | |  | **Cases** | **Controls** |  |  | | | |
| Yuan 2001 [20] | Cases 64.7, ctl 64.8 | M 831 | 524 of 831 ever/ current smokers | Shanghai China | n/a | | n/a | serum retinol | | -- | 209 | 622 | 12 y  (1986/89 to Sept 1998) | n | n | n/a | -- |
| Kumagai 1998 [21] | Median: lung CA cases 57, stomach CA 2 62, ctl 63 | M 100, F 21 | NR | Chinese | n/a | | n/a | serum vitA | | -- | 58 lung +22 stomach | 63 | 1 mo  (Nov-Dec 1995) | n | n | n/a | -- |
| Tominaga 1992 [22] | cases 51 (44-78), ctl 50 (43-80) | M 46, F 16 | NR | Japanese | n/a | | n/a | serum retinol | | -- | 31 | 31 | 19 mo (July 1988-Feb 1990) | n | n | n/a | -- |
| Kune 1989 [23] | Cases 67.0 (SD 6.2), ctl 66.6 (SD 8.5) | M 127 | NR | Australian | n/a | | n/a | serum vitA | | -- | 64 | 63 | 9 mo (June 1984-Feb 1985) | n | n | n/a | -- |
| Salonen 1985 [24] | range 30-64 | M 60, F 42 | NR (pop- based) | Finnish | n/a | | n/a | serum retinol | | -- | 51 | 51 | 4 y (1977- end of 1980) | n | n | n/a | -- |
| Hinds 1984 [25] | mean b/w 62-63 for all groups | M 705, F 286 | NR | Hawaiian | n/a | | n/a | serum retinol | | -- | 364 | 627 | 3 y (1979-1982) | n | n | n/a | -- |
| Wald 1980 [26] | range 35-64 | M 258 | NR | British cancer patients; 14 lung cancer | n/a | | n/a | serum retinol | | -- | 86 | 172 | 4 y (1975/8 to 1979) | n | n | n/a | -- |
| Knekt 1990 [27] | mean 57.4 +/-12.3 | M 1294, F 891 | 30-40% current smokers | Finnish | n/a | | n/a | serum retinol | | -- | 2185 | ENTIRE COHORT | 9 y (1968/72 -1977) | n | n | n/a | -- |
| Ito 2005 [28] | NR as mean or median | M 538, F 160 | 52-3% M current smokers; 3-7% F current smokers | Japanese | n/a | | n/a | serum reitnol | | -- | 147 | 311 | 11 y (1988/90 to 1999) | n | n | n/a | -- |
| *Ito 2003 [29]* | NR as mean or median | M 350, F 108 | 52-3% M current smokers, 3-7% M current smokers | Japanese | n/a | | n/a | serum retinol | | -- | 211 | 487 | 9 y (1988/90 to Dec 1997) | n | n | n/a | -- |
| LeGardeur 1990 [30] | 58 +/-7 | M114, F4 | NR | American | n/a | | n/a | serum retinol, RBP | | -- | 59 | NR | NR | n | n | n/a | -- |
| Connett 1989 [31] | Cases 49.2, ctl 48.9 | M 467 | NR | American men at high risk of CAD | n/a | | n/a | serum reitnol | | -- | 66 lung + 90 other | 311 | 10 y (1973-1983) | n | n | n/a | -- |
| Pastorino 1987 [32] | Cases 61.9 +/-10.1, ctl 51.5 +/-6.8 | F 206 | NR | Italian women | n/a | | n/a | serum retinol | | -- | 47 | 159 | NR | n | n | n/a | -- |
| Friedman 1986 [33] | mean 53.9 (range 26-78) | M 297, F 156 | NR | Californians | n/a | | n/a | serum retinol, RBP | | -- | 151 | 302 | 9 y (1988/90 to Dec 1997) | n | n | n/a | -- |
| Menkes 1986 [34] | 25-64 range (most) | M 202, F 93 | NR (pop-based) | Maryland | n/a | | n/a | serum retinol, RBP | | -- | 99 | 196 | 9 y (1974 to 1983) | n | n | n/a | -- |
| Darby 2001 [35] | ≤75 | M 534, F 285 | 829 of 2468 current smokers | British | n/a | | n/a | dietary and supplemental intake of preformed retinol | | -- | 982 | 1486 | 4 y (1988-93 | n | n | n/a | -- |
| Goodman 1996 [36] | range 45-74; 34% b/w 60-64 yrs | M 348, F 204 | 70% current, 30% former smokers | American: CARET cohort | n/a | | n/a | serum retinol | | -- | 276 | 276 | 12 y (1983/94 toDec 1995) | n | n | n/a | -- |
| **Observational – Cross sectional** | | | | | | | | | | | | | | | | | |
| Emri 2003 [Abstr] [37] | 44.8 +/-14 | M 82, F 78 | y | Turkish, w malignant mesothelioma | NR | | NR | serum retinol | | -- | 42 | 118 | n/a | n | n | n/a | -- |
| Sawicki 1985 [38] | NR | NR | NR | Polish | NR | | NR | serum vitA | | -- | 20 | 20 | n/a | n | n | n/a | -- |
| Jain 1984 [39] | NR | NR | NR | East Indian | NR | | NR | serum retinol | | -- | 25 | 25 | n/a | n | n | n/a | -- |
| Atukorala 1979 [40] | Cases 64.7 (46-82), ctl160.3 (47-74), ctl2 63.4 (48-75) | M 37, F 10 | NR | British: cases, controls with other lung disease, or ctl w other CAs | NR | | NR | serum retinol, RBP | | -- | 26 | 21 | n/a | n | n | n/a | -- |
| Basu 1976 [41] | Cases 67 (48-78), ctl1 61 (53-70), ctl2 58 (49-68) | NR | y | British | NR | | NR | serum retinol | | -- | 28 | 19 | n/a | n | n | n/a | -- |
| Cohen 1978 [42] | 59 (40-83) | M 64, F 3 | avg smoking history 30 pack-years | American: Washington DC | NR | | none | serum retinol | | -- | 67 | NR | n/a | n | n | n/a | -- |
| Moulas 2006 [43] | Cases 66.2 +/- 12.3; Ctl 44.5 +/-16.7 | M 35, F 1 | NR | Greek | NR | | none | serum retinol, retinoic acid, and retinyl palmitate | | -- | 36 | 27 | n/a | n | n | n/a | -- |
| Gackowski 2005 [44] | Cases 64 (44-83); ctl 59 (40-87) | M 100, F 24 | smokers and nonsmokers in both case and ctl groups | Polish | NR | | none | serum retinol, retinoic acid, and retinyl palmitate | | -- | 67 | 57 | n/a | n | n | n/a | -- |
| Mura 1987 [Abstr] [45] | 40-75 range | M 252, F 0 | NR | French | NR | | NR | serum retinol | | -- | 70 lung +42 other | 140 | n/a | n | n | n/a | -- |

**References**

1. Recchia F, Saggio G, Cesta A, Alesse E, Gallo R, et al. (2005) Phase II randomized study of interleukin-2 with or without 13-cis retinoic acid as maintenance therapy in patients with advanced cancer responsive to chemotherapy. Anticancer Res 25: 3149-3158.

2. Ramlau R, Zatloukal P, Jassem J, Schwarzenberger P, Orlov SV, et al. (2008) Randomized phase III trial comparing bexarotene (L1069-49)/cisplatin/ vinorelbine with cisplatin/ vinorelbine in chemotherapy-naïve patients with advanced or metastatic non-small-cell lung cancer: SPIRIT I. J Clin Oncol 26: 1886-1892.

3. Blumenschein GR, Khuri FR, Von Pawel J, Gatzemeier U, Miller WH, et al (2008) Phase III trial comparing carboplatin, paclitaxel, and bexarotene with carboplatin and paclitaxel in chemotherapy –naïve patients with advanced or metastatic non-small cell lung cancer: SPIRIT II. J Clin Oncol 26: 1879-1885.

4. Ruotsalainen T, Halme M, Isokangas OP, Pyrhönen S, Mäntylä M, et al. (2000) Interferon-alpha and 13-cis-retinoic acid as maintenance therapy after high-dose combination chemotherapy with growth factor support for small cell lung cancer – a feasibility study. Anti-Cancer Drugs 11: 101-108.

5. Rizvi N, Hawkins MJ, Eisenberg PD, Yocum RC, Reich SD (2001) Placebo-controlled trial of bexarotene, a retinoid X receptor agonist, as maintenance therapy for patients treated with chemotherapy for advanced non-small-cell lung cancer. Clinical Lung Cancer 2: 210-215.

6. Omenn GS, Goodman GE, Thornquist MD, Balmes J, Cullen MR, et al. (1996) Effects of a combination of beta carotene and vitamin A on lung cancer and cardiovascular disease. N Engl J med 334: 1150-1155.

7. de Klerk NH, Musk AW, Ambrosini GL, Eccles JL, Hansen J, et al. (1998) Vitamin A and cancer prevention II: comparison of the effects of retinol and beta-carotene. Int J Cancer 75: 362-367.

8. Kamangar F, Qiao YL, Yu B, Sun XD, Abnet CC, et al. (2006) Lung cancer chemoprevention: a randomized, double-blind trial in Linxian, China. Cancer Epidemiol Biomarkers Prev 15: 1562-1564.

9. Pastorino U, Infante M, Maioli M, Chiesa G, Buyse M, et al. (1993) Adjuvant treatment of stage I lung cancer with high-dose vitamin A. J Clin Oncol 11: 1216-1222.

10. van Zandwijk N, Dalesio O, Pastorino U, de Vries N, van Tinteren H (2000) EUROSCAN, a randomized trial of vitamin A and N-acetylcysteine in patients with head and neck cancer or lung cancer. For the European Organization for Research and Treatment of Cancer Head and Neck and Lung Cancer Cooperative Groups. J Natl Cancer Inst 92: 977-986.

11. Lippman SM, Lee JJ, Karp DD, Vokes EE, Venner SE, et al. (2001) Randomized phase III intergroup trial of isotretinoin to prevent second primary tumors in stage I non-small-cell lung cancer. J Natl Cancer Inst 93: 605-617.

12. Alfonso HS, Fritschi L, de Klerk NH, Ambrosini GL, Beilby J, et al. (2006) Plasma vitamin concentrations and incidence of mesothelioma and lung cancer in individuals exposed to crocidolite at Wittenoom, Western Australia. Eur J Cancer Prev 15: 290-294.

13. Holick CN, Michaud DS, Stolzenberg-Solomon R, Mayne ST, Pietinen P, et al. (2002) Dietary carotenoids, serum beta-carotene, and retinol and risk of lung cancer in the alpha-tocopherol, beta-carotene cohort study. Am J Epidemiol 156: 536-547.

14. Stahelin HB, Gey KF, Eichholzer M, Ludin E, Bernasconi F, et al. (1991) Plasma antioxidant vitamins and subsequent cancer mortality in the 12-year follow-up of the prospective Basel Study. Am J Epidemiol 133: 766-775.

15. Ito Y, Kurata M, Hioki R, Suzuki K, Ochiai J, et al. (2005) Cancer mortality and serum levels of carotenoids, retinol, and tocopherol: a population –based follow-up study of inhabitants fo a rural area of Japan. Asian Pac J Cancer Prev 6: 10-15.

16. Ito Y, Suzuki K, Ishii J, Hisida H, Tamakoshi A, et al. (2006) A population-based follow-up study on mortality from cancer or cardiovascular disease and serum carotenoids, retinol and tocopherols in Japanese inhabitants. Asian Pac J Cancer Prev 7: 533-546.

17. Yong LC, Brown CC, Schatzkin A, Dresser CM, Slesinski MJ, et al. (1997) Intake of vitamins E, C, and A and risk of lung cancer. The NHANES I epidemiologic followup study. First National Health and Nutrition Examination Survey. Am J Epidemiol 146: 231-243.

18. Paganini-Hill A, Chao A, Ross RK, Henderson BE (1987) Vitamin A, beta-carotene, and the risk of lung cancer: a prospective study. J Natl Cancer Inst 79: 443-448.

19. Satia JA, Littman A, Slatore CG, Galanko JA, White E (2009) Long-term use of beta-carotene, retinol, lycopene, and lutein supplements and lung cancer risk: results from the VITamins And Lifestyle (VITAL) study. Am J Epidemiol 169: 815-828.

20. Yuan JM, Ross RK, Chu XD, Gao YT, Yu MC (2001) Prediagnostic levels of beta-crytoxanthin and retinol predict smoking-related lung cancer risk in Shanghai, china. Cancer Epidemiol Biomarkers Prev 10: 767-773.

21. Kumagai Y, Pi JB, Lee S, Sun GF, Yamanushi T, et al. (1998) Serum antioxidant vitamins and risk of lung and stomach cancers in Shenyang, China. Cancer Lett 129: 145-149.

22. Tominaga K, Saito Y, Mori K, Miyazawa N, Yokoi K, et al. (1992) An evaluation of serum microelement concentrations in lung cancer and matched non-cancer patients to determine the risk of developing lung cancer: a preliminary study. Jpn J Clin Oncol 22: 96-101.

23. Kune GA, Kune S, Watson LF, Pierce R, Field B, et al. (1989) Serum levels of beta-carotene, vitamin A, and zinc in male lung cancer cases and controls. Nutr Cancer 12: 169-176.

24. Salonen JT, Salonen R, Lappetelainen R, Maenpaa PH, Alfthan G, et al. (1985) Risk of cancer in relation to serum concentrations of selenium and vitamins A nd E: matched case-control analysis of prospective data. Br med J (Clin Res Ed) 290: 417-420.

25. Hinds MW, Kolonel LN, Hankin JH, Lee J (1984) Dietary vitamin A, carotene, vitamin C and risk oflung cancer in Hawaii. Am J Epidemiol 119: 227-237.

26. Wald N, Idle M, Boreham J, Bailey A (1980) Low serum-vitamin A and subsequent risk of cancer. Preliminary results of a prospective study. Lancet 2: 813-815.

27. Knekt P, Aromaa A, Maatela J, Aaran RK, Nikkari T, et al. (1990) Serum vitamin A and subsequent risk of cancer: cancer incidence follow-up of the Finnish Mobile Clinic Health Examination Survey. Am J Epidemiol 132: 857-870.

28. Ito Y, Wakai K, Suzuki K, Ozasa K, Watanabe Y, et al. (2005) Lung cancer mortality and serum levels of carotenoids, retinol, tocopherols, and folic acid in mena nd women: a case-control study nested in the JACC Study. J Epidemiol 15 Suppl 2: S140-149.

29. Ito Y, Wakai K, Suzuki K, Tamakoshi A, Seki N, et al. (2003) Serum carotenoids and mortality from lung cancer: a case-control study nested in the Japan Collaborative Cohort (JACC) study. Cancer Sci 94: 57-63.

30. LeGardeur BY, Lopez A, Jonson WD (1990) A case-control study of serum vitamins A, E, and C in lung cancer patients. Nutr Cancer 14: 133-140.

31. Connett JE, Kuller LH, Kjelsberg MO, Polk BF, Collins G, et al. (1989) Relationship between carotenoids and cancer. The Multiple Risk Factor Intervention Trial (MRFIT) Study. Cancer 64: 126-134.

32. Pastorino U, Pisani P, Berrino F, Andreoli C, Barbieri A, et al. (1987) Vitamin A and female lung cancer: a case-control study on plasma and diet. Nutr Cancer 10: 171-179.

33. Friedman GD, Blaner WS, Goodman DS, Vogelman JH, Brind JL, et al. (1986) Serum retinol and retinol-binding protein levels do not predict subsequent lung cancer. Am J Epidemiol 123: 781-789.

34. Menkes MS, Comstock GW, Vuilleumier JP, Helsing KJ, Rider Aa, et al. (1986) Serum beta-carotene, vitamins A and E, selenium, and the risk of lung cancer. N Engl J med 315: 1250-1254.

35. Darby S, Whitley E, Doll R, Key T, Silcocks P (2001) Diet, smoking and lung cancer: a case-control study of 1000 cases and 1500 controls in South-West England. Br J Cancer 84: 728-735.

36. Goodman GE, Thornquist M, Kestin M, Metch B, Anderson G, et al. (1996) The association between participant characteristics and serum concentrations of beta-carotene, retinol, reninyl palmitate, and alpha tocopherol among participants in the Carotene and Retinol Efficacy Trial (CARET) for prevention of lung cancer. Cancer Epidemiol Biomarkers Prev 5: 815-821.

37. Emri S, Kilickap S, Kadilar C, Khalil M, Akay H, et al. (2003) P-510 Serum vitamin E, vitamin C, beta carotene, and retinol levels and the risk of malignant pleural mesothelioma in Turkey. Lung Cancer 41: S219.

38. Sawicki J, Ostrowski J, Swietochowska B, Janik P, Sikora J, et al. (1985) Vitamin A (retinol) level in colon and lung cancer patient sera. Neoplasma 32: 225-227.

39. Jain NC, Gupta AP, Saksena HC (1984) Serum vitamin ‘A’ in bronchogenic carcinoma. J Assoc Physicians India 32: 485-486.

40. Atukorala S, Basu TK, Dickerson JW, Donaldson D, Sakula A (1979) Vitamin A, zinc and lung cancer. Br J Cancer 40: 927-931.

41. Basu TK, Donaldson D, Jenner M, Williams DC, Sakula A (1976) Plasma vitamin A in patients with bronchial carcinoma. Br J Cancer 33: 119-121.

42. Cohen MH, Primack A, Broder LE, Williams LR (1978) Vitamin A serum and dietary vitamin A intake in lung cancer patients. Cancer Lett 4: 51-54.

43. Moulas AN, Gerogianni IC, Papadopoulos D, Gourgoulianis KI (2006) Serum retinoic acid, retinol and retinyl palmitate levels in patients with lung cancer. Respirology 11: 169-174.

44. Gackowski D, Kowalewski J, Siomek A, Olinski R (2005) Oxidative DNA damage and antioxidant vitamin level: comparison among lung cancer patients, healthy smokers and nonsmokers. Int J Cancer 114: 153-156.

45. Mura P, Castel O, Gros N, Suire R (1987) Vitamin A concentrations in lung cancer. Clin Chem 33: 716.
