# Supplementary material for: Vitamin A and Retinoid Derivatives for Lung Cancer: A Systematic Review and Meta Analysis
Source: PLoS One. 2011 Jun 27;6(6):e21107. doi: 10.1371/journal.pone.0021107 (PMC3124481; doi:10.1371/journal.pone.0021107)
Supplement: Table S5 — Outcomes of Controlled Human Studies for Vitamin A and Lung Cancer. 95% CI 95% confidence interval; Ad adenocarcinoma; Adj adjusted; AHR adjusted hazard ratio; AOR adjusted odds ratio; C control group; CR complete response; DS disease stabilization; DSR disease stabilization rate; EC epidermoid carcinoma; HR hazard ratio; LC large cell carcinoma; mo months; NR not reported; NS not significant; OR odds ratio; PD progressive disease; PR partial response; RR relative risk; SmC small cell carcinoma; T treatment group. (DOC) [file pone.0021107.s005.doc]

**Table S5. Outcomes of Controlled Human Studies for Vitamin A and Lung Cancer**

| **Ref** | **Median Survival (mo)** | | | | **Progression Free Survival (mo)** | | | | **Response Rate** | | | | **Additonal Survival Measures** | | | | | | **Other** | | | | | | | | | |  | |
| --- | --- | --- | --- | --- | --- | --- | --- | --- | --- | --- | --- | --- | --- | --- | --- | --- | --- | --- | --- | --- | --- | --- | --- | --- | --- | --- | --- | --- | --- | --- |
| **RCTs** **Treatment** | T1 | T2 | C | p value | T1 | T2 | C | p value | T1 | T2 | C | p value | Outcome | T1 | T2 | C | p value | | Outcome | | T1 | | T2 | | | C | | p value | |  |
| Recchia 2005 [1] | not reached | -- | -- | -- | 28.45 | -- | 12.35 | 0.19 | -- | -- | -- | -- | -- | -- | -- | -- | -- | | Immune Parameters | | NK cells 0.0451 | | Lymph. 0.0353 | | | CD4+/CD8+0.115 | | VEGF 0.0020 | |  |
| Ramlau 2008 [2] | 8.7 | -- | 9.9 | 0.30 | 4.3 | -- | 5.0 | 0.95 | 16.70% | -- | 24.40% | 0.224 | Projected 2yr survival | 13.20% | -- | 15.70% | -- | | DSR | | 37% | | -- | | | 35.30% | | -- | |  |
|  |  |  |  |  |  |  |  |  |  |  |  |  |  | 41/ 311 | -- | 49/ 312 | -- | |  | | 115/ 311 | | -- | | | 110/ 312 | | -- | |  |
| Blumenschein 2008 [3] | 8.5 | -- | 9.2 | 0.20 | 4.1 | -- | 4.9 | 0.06 | 19.30% | -- | 23.50% | 0.24 | Projected 2yr survival | 12.40% | -- | 16.30% | 0.20 | | DSR | | 40.20% | | -- | | | 37.60% | | -- | |  |
|  |  |  |  |  |  |  |  |  |  |  |  |  |  | 38/ 308 | -- | 50/ 306 | -- | |  | | 123/ 306 | | -- | | | 115/ 306 | | -- | |  |
| Ruotsalainen 2000 [4] | 17.7 | 13.5 | 16.2 | 0.79 | 6.5 | 5.5 | 4.7 | 0.24 | CR5, PR10, PD0, DS2= 15/17 | CR7, PR16, PD0, DS0= 23/23 | CR8, PR10, PD0, DS2= 18/20 | -- | 1 –yr survival | 82% | 56% | 55% | 0.18 | | Time to Replapse (mo) | | 8.6 | | 8.0 | | | 6.8 | | 0.31 | |  |
|  |  |  |  |  |  |  |  |  | 88% | 100% | 90% | -- |  | 14/ 17 | 13/ 23 | 11/ 20 | -- | |  | | -- | | -- | | | -- | | -- | |  |
| Rizvi 2001 [5] | 328 d | 587 d | 537 d | NS | -- | -- | -- | -- | No objective response | -- | -- | -- | Survival at data lock | 6/ 21 | 7/ 15 | 5/ 16 | -- | | Time to Replapse (mo) | | 11.7 | | 18.3 | | | 8.0 | | 0.056 | |  |
| **Primary Prevention** | **Lung Cancer Incidence** | | | | **Mesothelioma Incidence** | | | | **All Cause Mortality** | | | |  |  |  |  |  | |  | |  | |  | | |  | |  | |  |
|  | Measure | T | C | p value/ 95%CI | Measure | T | C | p value/ 95%CI | Measure | T | C | p value/ 95%CI |  |  |  |  |  | |  | |  | |  | | |  | |  | |  |
| Omenn 1996 (CARET) [6] | Lung cancer/ person-yr of f/u | 229/ 38,684 | 159/ 34,450 | -- | -- | -- | -- | -- | Deaths/ 1000 person-yr | 14.45 | 11.91 | -- |  |  |  |  |  | |  | |  | |  | | |  | |  | |  |
|  | Incidence rate x1000 (95%CI) | 5.9 (5.2-6.7) | 4.6 (4.0-5.4) | -- | -- | -- | -- | -- | -- | -- | -- | -- |  |  |  |  |  | |  | |  | |  | | |  | |  | |  |
|  | RR | 1.28 | -- | 1.04-1.57 | -- | -- | -- | -- | RR | 1.17 | -- | 1.03-1.33 |  |  |  |  |  | |  | |  | |  | | |  | |  | |  |
| deKlerk 1998 [7] | No. cases | 3/ 512 | 6/ 512 | -- | No. cases | 4/ 512 | 12/ 512 | -- | No. cases | 21/ 512 | 37/ 512 | -- |  |  |  |  |  | |  | |  | |  | | |  | |  | |  |
|  | RR | 0.66 | -- | 0.19-2.32 | RR | 0.24 | -- | 0.07-0.86 | RR | 0.65 | -- | 0.33-0.95 |  |  |  |  |  | |  | |  | |  | | |  | |  | |  |
| Kamangar 2006 [8] | RR (Retinol+ zinc group) | 1.16 | -- | 0.84-1.60 | -- | -- | -- | -- | -- | -- | -- | -- |  |  |  |  |  | |  | |  | |  | | |  | |  | |  |
| **Secondary Prevention** | **Event Free Survival** | | | | **Second Primary Tumor (SPT)** | | | | **Recurrence** | | | | **5yr** **Survival** | | | | | | **Death** | | | | | | | | | | |  |
|  | Measure | T | C | p value | Measure | T | C | p value | Measure | T | C | p value | Measure | T | C | p value |  | | Measure | | T | | C | | | p value | |  | |  |
| Pastorino 1993 [9] | Disease free at 5yr | 64% | 51% | 0.054 | New primary tumor at any site (estimatedat 5yr) | 14% | 24% | 0.09 | New primary tumor in field of chemoprevention (lung, HN, bladder) | 11% | 20% | 0.45 | (Estimated at 5yr) | 62% | 54% | 0.44 | -- | | Death at 5yr | | 55/ 150 | | 64/ 157 | | | 0.44 | | -- | |  |
|  |  | 94/ 150 | 82/ 157 | “ |  | 18/ 150 | 29/ 157 | “ |  | 13/ 150 | 25/ 157 | “ |  | 95/ 150 | 93/ 157 | “ | -- | |  |  |  |  |  |  |  |  |  |  |  |  |
| van Zandwijk 2000 [10] | Retinyl vs no retinyl | 565/ 1290 | 451/ 1283 | -- | Retinyl vs no retinyl | 115/ 1290 | 93/ 1283 | -- | Local recurrence: Retinyl vs no retinyl | 170/ 1290 | 161/ 1283 | -- | Retinyl vs no retinyl | 318/ 1290 | 314/ 1284 | -- | -- | | Death at 5yr: Retinyl vs no retinyl | | 64/ 1290 | | 69/ 1283 | | | -- | | -- | |  |
|  | Retinyl vs no treatment | -- | -- | -- | Retinyl vs no treatment | 61/ 647 | 32/ 641 | -- | Retinyl vs no treatment | 92/ 647 | 93/ 641 | -- | Retinyl vs no treatment | 612/ 647 | 610/ 641 | -- | -- | | Retinyl vs no treatment | | 35/ 647 | | 31/ 641 | | | -- | | -- | |  |
| Lippman 2001 [11] | -- | -- | -- | -- | Event rate (# cases/ total # follow up years, 95%CI) | 4.2 (3.3-5.2) | 3.9 (3.1-4.9) | -- | Event rate (# cases/ total # follow up years, 95%CI) | 6.2 (5.1-7.5) | 6.2 (5.2-7.5) | -- | -- | -- | -- | -- | -- | | Event rate (# cases/ total # follow up years, 95%CI) | | 7.5 (6.4-8.8) | | 7.1 (6.0-8.4) | | | -- | | -- | |  |
| **Observational**  **Prospective Cohorts** | **Risk of lung cancer** | | | | | | | | **Risk of mesothelioma** | | | | | | | | |  | |  | |  | |  |  | |  | | | |
|  | Unadjusted Effect Measure | Value | 95% CI | -- | Adjusted Effect Measure | Value | 95% CI |  | Unadjusted Effect Measure | Value | 95% CI | -- | Adjusted Effect Measure | Value | 95% CI |  |  | |  | |  | |  | | |  | |  | |  |
| Alfonso 2006 [12] | -- |  |  |  | Multivariate HR (first measurement) | 0.90 | 0.54-1.51 |  | -- |  |  |  | Multivariate HR (first measurement) | 0.63 | 0.41-0.99 |  |  | |  | |  | |  | | |  | |  | |  |
|  | -- |  |  |  | Multivariate HR (every visit) | 0.76 | 0.50-1.17 |  | -- |  |  |  | Multivariate HR (every visit) | 0.71 | 0.51-1.00 |  |  | |  | |  | |  | | |  | |  | |  |
| Holick 2002 [13] | Age adjusted (only) RR | 0.68 | 0.58-0.80 |  | Multivariate RR | 0.73 | 0.62-0.86 |  |  |  |  |  |  |  |  |  |  | |  | |  | |  | | |  | |  | |  |
| Stahelin 1991 [14] | -- |  |  |  | Adj RR Bronchus cancer: age >60 | 2.54 | 1.45-4.43 |  |  |  |  |  |  |  |  |  |  | |  | |  | |  | | |  | |  | |  |
|  |  |  |  |  | age ≤60 | 1.15 | 0.47-2.81 |  |  |  |  |  |  |  |  |  |  | |  | |  | |  | | |  | |  | |  |
| Ito 2005, 2006 [15,16] | -- |  |  |  | AHR | 0.46 | 0.16-1.43 |  |  |  |  |  |  |  |  |  |  | |  | |  | |  | | |  | |  | |  |
|  | -- |  |  |  | AHR | 0.56 | 0.20-1.51 |  |  |  |  |  |  |  |  |  |  | |  | |  | |  | | |  | |  | |  |
| Yong 1997 [17] | NS effects; data not shown |  |  |  | -- |  |  |  |  |  |  |  |  |  |  |  |  | |  | |  | |  | | |  | |  | |  |
| Paganini-Hill 1987 [18] | -- |  |  |  | Age-adj incidence rates | 1.43 | n/a |  |  |  |  |  |  |  |  |  |  | |  | |  | |  | | |  | |  | |  |
| Satia 2009 [19] | -- |  |  |  | AHR (10-yr avd daily dose) | 1.25 | 0.93-1.69 |  |  |  |  |  |  |  |  |  |  | |  | |  | |  | | |  | |  | |  |
| **Observational**  **Case Control Studies** | **Risk of lung cancer** | | | | | | | | **Risk of mesothelioma** | | | | | | | | |  | |  | |  | |  |  | |  | | | |
|  | Unadjusted Effect Measure | Value | 95% CI | -- | Adjusted Effect Measure | Value | 95% CI |  | Unadjusted Effect Measure | Value | 95% CI | -- | Adjusted Effect Measure | Value | 95% CI |  |  | |  | |  | |  | | |  | |  | |  |
| Yuan 2001 [20] | OR | 0.69 | 0.44-1.09 |  | AOR | 0.65 | 0.37-1.09 |  |  |  |  |  |  |  |  |  |  | |  | |  | |  | | |  | |  | |  |
| Kumangai 1998 [21] | OR (serum vitA<0.52 ug/ml) | 3.7 | 1.5-9.0 |  | -- |  |  |  |  |  |  |  |  |  |  |  |  | |  | |  | |  | | |  | |  | |  |
| Tominaga 1992 [22] | RR | 5.94 | 1.20-29.34 |  | -- |  |  |  |  |  |  |  |  |  |  |  |  | |  | |  | |  | | |  | |  | |  |
| Kune 1989 [23] | NR |  |  |  | -- |  |  |  |  |  |  |  |  |  |  |  |  | |  | |  | |  | | |  | |  | |  |
| Salonen 1985 [24] | -- |  |  |  | Adj RR (lower vs higher s-retinol) | 0.9 | 0.3-2.5 |  |  |  |  |  |  |  |  |  |  | |  | |  | |  | | |  | |  | |  |
| Hinds 1984 [25] | -- |  |  |  | AOR | 1.4 | 0.9-2.2 |  |  |  |  |  |  |  |  |  |  | |  | |  | |  | | |  | |  | |  |
| Wald 1980 [26] | RR cancer at all sites | 0.67 | NR  p trend <0.025 |  | -- |  |  |  |  |  |  |  |  |  |  |  |  | |  | |  | |  | | |  | |  | |  |
| Knekt 1990 [27] | -- |  |  |  | Smoking adj RR men | 1.4 | 0.9-2.1 |  |  |  |  |  |  |  |  |  |  | |  | |  | |  | | |  | |  | |  |
|  | -- |  |  |  | Smoking adj RR women | 1.5 | 0.9-2.3 |  |  |  |  |  |  |  |  |  |  | |  | |  | |  | | |  | |  | |  |
| Ito 2005, 2003 [28,29] | -- |  |  |  | AOR men | 0.49 | 0.22-1.08 |  |  |  |  |  |  |  |  |  |  | |  | |  | |  | | |  | |  | |  |
|  | -- |  |  |  | AOR women | 2.25 | 0.68-7.47 |  |  |  |  |  |  |  |  |  |  | |  | |  | |  | | |  | |  | |  |
|  | -- |  |  |  | AOR | 1.02 | 0.49-2.14 |  |  |  |  |  |  |  |  |  |  | |  | |  | |  | | |  | |  | |  |
| LeGardeur 1990 [30] | NR |  |  |  |  |  |  |  |  |  |  |  |  |  |  |  |  | |  | |  | |  | | |  | |  | |  |
| Connett 1989 [31] | NR |  |  |  |  |  |  |  |  |  |  |  |  |  |  |  |  | |  | |  | |  | | |  | |  | |  |
| Pastorino 1987 [32] | Crude OR | 1.95 | NR; trend NS |  | AOR, multivariate | 1.13 | NR; trend NS |  |  |  |  |  |  |  |  |  |  | |  | |  | |  | | |  | |  | |  |
| Friedman 1986 [33] | OR | 1.2 | NR |  | NR |  |  |  |  |  |  |  |  |  |  |  |  | |  | |  | |  | | |  | |  | |  |
| Menkes 1986 [34] | -- |  |  |  | OR | 1.13 | NR; X2 trend (1degree freedom) 0.17 |  |  |  |  |  |  |  |  |  |  | |  | |  | |  | | |  | |  | |  |
| Darby 2001 [35] | -- |  |  |  | Adj RR | 1.61 | 1.24-2.10 |  |  |  |  |  |  |  |  |  |  | |  | |  | |  | | |  | |  | |  |
| Goodman 2003 [36] | -- |  |  |  | AOR, retinol | 0.69 | 0.42-1.14 |  |  |  |  |  |  |  |  |  |  | |  | |  | |  | | |  | |  | |  |
|  | -- |  |  |  | AOR retinyl palmitate | 0.73 | 0.42-1.28 |  |  |  |  |  |  |  |  |  |  | |  | |  | |  | | |  | |  | |  |
| **ObservationalCross-sectional** | **Effect sizes were not calculated in cross-sectional studies; comparisons of vitA status conducted only** | | | | | | |  |  |  |  |  |  |  |  |  |  | |  | |  | |  | | |  | |  | |  |
|  | **Measure** | **Cases** | **Controls1** | **Controls 2** | **Controls 3** | **p value** | **units** |  |  |  |  |  |  |  |  |  |  | |  | |  | |  | | |  | |  | |  |
| Emri 2003 [Abstr] [37] | serum retinol | 2.04 +/-0.03 | 2.53 +/- 0.03 | 2.09 +/- 0.04 | 2.54 +/- 0.05 | NR | µmol/L |  |  |  |  |  |  |  |  |  |  | |  | |  | |  | | |  | |  | |  |
| Sawicki 1985 [38] | serum retinol | 40.0 +/-9.1 | 75.4 +/-11.2 | -- | -- | NR | NR – likely µg/dL |  |  |  |  |  |  |  |  |  |  | |  | |  | |  | | |  | |  | |  |
| Jain 1984 [39] | serum retinol | 23.6 +/-12.6 | 37.9 +/-10.3 | -- | -- | p<0.001 | µg/dL |  |  |  |  |  |  |  |  |  |  | |  | |  | |  | | |  | |  | |  |
| Atukorala 1979 [40] | serum retinol | 46.9 +/-2.4 | 58.3 +/-1.6 | 61.9 +/-2.2 | -- | p<0.01, <0.001 resp | µg/dL |  |  |  |  |  |  |  |  |  |  | |  | |  | |  | | |  | |  | |  |
| Basu 1976 [41] | serum retinol | 45.6 +/-5.8 | 64.3 +/-4.6 | 68.4 +/-5.0 | -- | p<0.01 | µg/dL |  |  |  |  |  |  |  |  |  |  | |  | |  | |  | | |  | |  | |  |
| Cohen 1978 [42] | serum retinol | EC 58 +/-16; SmC58 +/-15; Ad 58 +/-17; LC50 +/-12 | 50 +/-20 | -- | -- | NR | µg/dL |  |  |  |  |  |  |  |  |  |  | |  | |  | |  | | |  | |  | |  |
| Moulas 2006 [43] | serum retinol | 733.5 +/- 326.4 | 734.5 +/- 337.1 | -- | -- | NS | ng/mL |  |  |  |  |  |  |  |  |  |  | |  | |  | |  | | |  | |  | |  |
|  | serum retinoic acid | 1.9 +/- 0.6 | 2.5 +/- 1.1 | -- | -- | p<0.05 | ng/mL |  |  |  |  |  |  |  |  |  |  | |  | |  | |  | | |  | |  | |  |
|  | serum retinyl palmitate | 14.3 +/- 9.7 | 16.7 +/- 13.7 | -- | -- | NS | ng/mL |  |  |  |  |  |  |  |  |  |  | |  | |  | |  | | |  | |  | |  |
| Gackowski 2005 [44] | serum retinol | smokers 1.25 +/-0.73; ex-smokers 1.05 +/-0.39 | smokers 1.33 +/-0.40; ex-smokers 1.26 +/-0.63 | -- | -- | NS | µM |  |  |  |  |  |  |  |  |  |  | |  | |  | |  | | |  | |  | |  |
| Mura 1987 [45] | serum retinol | 0.82 +/-0.52 | 0.93 +/-0.40 | 0.78 +/-42 | -- | NR | mg/L |  |  |  |  |  |  |  |  |  |  | |  | |  | |  | | |  | |  | |  |

**References**

1. Recchia F, Saggio G, Cesta A, Alesse E, Gallo R, et al. (2005) Phase II randomized study of interleukin-2 with or without 13-cis retinoic acid as maintenance therapy in patients with advanced cancer responsive to chemotherapy. Anticancer Res 25: 3149-3158.

2. Ramlau R, Zatloukal P, Jassem J, Schwarzenberger P, Orlov SV, et al. (2008) Randomized phase III trial comparing bexarotene (L1069-49)/cisplatin/ vinorelbine with cisplatin/ vinorelbine in chemotherapy-naïve patients with advanced or metastatic non-small-cell lung cancer: SPIRIT I. J Clin Oncol 26: 1886-1892.

3. Blumenschein GR, Khuri FR, Von Pawel J, Gatzemeier U, Miller WH, et al (2008) Phase III trial comparing carboplatin, paclitaxel, and bexarotene with carboplatin and paclitaxel in chemotherapy –naïve patients with advanced or metastatic non-small cell lung cancer: SPIRIT II. J Clin Oncol 26: 1879-1885.

4. Ruotsalainen T, Halme M, Isokangas OP, Pyrhönen S, Mäntylä M, et al. (2000) Interferon-alpha and 13-cis-retinoic acid as maintenance therapy after high-dose combination chemotherapy with growth factor support for small cell lung cancer – a feasibility study. Anti-Cancer Drugs 11: 101-108.

5. Rizvi N, Hawkins MJ, Eisenberg PD, Yocum RC, Reich SD (2001) Placebo-controlled trial of bexarotene, a retinoid X receptor agonist, as maintenance therapy for patients treated with chemotherapy for advanced non-small-cell lung cancer. Clinical Lung Cancer 2: 210-215.

6. Omenn GS, Goodman GE, Thornquist MD, Balmes J, Cullen MR, et al. (1996) Effects of a combination of beta carotene and vitamin A on lung cancer and cardiovascular disease. N Engl J med 334: 1150-1155.

7. de Klerk NH, Musk AW, Ambrosini GL, Eccles JL, Hansen J, et al. (1998) Vitamin A and cancer prevention II: comparison of the effects of retinol and beta-carotene. Int J Cancer 75: 362-367.

8. Kamangar F, Qiao YL, Yu B, Sun XD, Abnet CC, et al. (2006) Lung cancer chemoprevention: a randomized, double-blind trial in Linxian, China. Cancer Epidemiol Biomarkers Prev 15: 1562-1564.

9. Pastorino U, Infante M, Maioli M, Chiesa G, Buyse M, et al. (1993) Adjuvant treatment of stage I lung cancer with high-dose vitamin A. J Clin Oncol 11: 1216-1222.

10. van Zandwijk N, Dalesio O, Pastorino U, de Vries N, van Tinteren H (2000) EUROSCAN, a randomized trial of vitamin A and N-acetylcysteine in patients with head and neck cancer or lung cancer. For the European Organization for Research and Treatment of Cancer Head and Neck and Lung Cancer Cooperative Groups. J Natl Cancer Inst 92: 977-986.

11. Lippman SM, Lee JJ, Karp DD, Vokes EE, Venner SE, et al. (2001) Randomized phase III intergroup trial of isotretinoin to prevent second primary tumors in stage I non-small-cell lung cancer. J Natl Cancer Inst 93: 605-617.

12. Alfonso HS, Fritschi L, de Klerk NH, Ambrosini GL, Beilby J, et al. (2006) Plasma vitamin concentrations and incidence of mesothelioma and lung cancer in individuals exposed to crocidolite at Wittenoom, Western Australia. Eur J Cancer Prev 15: 290-294.

13. Holick CN, Michaud DS, Stolzenberg-Solomon R, Mayne ST, Pietinen P, et al. (2002) Dietary carotenoids, serum beta-carotene, and retinol and risk of lung cancer in the alpha-tocopherol, beta-carotene cohort study. Am J Epidemiol 156: 536-547.

14. Stahelin HB, Gey KF, Eichholzer M, Ludin E, Bernasconi F, et al. (1991) Plasma antioxidant vitamins and subsequent cancer mortality in the 12-year follow-up of the prospective Basel Study. Am J Epidemiol 133: 766-775.

15. Ito Y, Kurata M, Hioki R, Suzuki K, Ochiai J, et al. (2005) Cancer mortality and serum levels of carotenoids, retinol, and tocopherol: a population –based follow-up study of inhabitants fo a rural area of Japan. Asian Pac J Cancer Prev 6: 10-15.

16. Ito Y, Suzuki K, Ishii J, Hisida H, Tamakoshi A, et al. (2006) A population-based follow-up study on mortality from cancer or cardiovascular disease and serum carotenoids, retinol and tocopherols in Japanese inhabitants. Asian Pac J Cancer Prev 7: 533-546.

17. Yong LC, Brown CC, Schatzkin A, Dresser CM, Slesinski MJ, et al. (1997) Intake of vitamins E, C, and A and risk of lung cancer. The NHANES I epidemiologic followup study. First National Health and Nutrition Examination Survey. Am J Epidemiol 146: 231-243.

18. Paganini-Hill A, Chao A, Ross RK, Henderson BE (1987) Vitamin A, beta-carotene, and the risk of lung cancer: a prospective study. J Natl Cancer Inst 79: 443-448.

19. Satia JA, Littman A, Slatore CG, Galanko JA, White E (2009) Long-term use of beta-carotene, retinol, lycopene, and lutein supplements and lung cancer risk: results from the VITamins And Lifestyle (VITAL) study. Am J Epidemiol 169: 815-828.

20. Yuan JM, Ross RK, Chu XD, Gao YT, Yu MC (2001) Prediagnostic levels of beta-crytoxanthin and retinol predict smoking-related lung cancer risk in Shanghai, china. Cancer Epidemiol Biomarkers Prev 10: 767-773.

21. Kumagai Y, Pi JB, Lee S, Sun GF, Yamanushi T, et al. (1998) Serum antioxidant vitamins and risk of lung and stomach cancers in Shenyang, China. Cancer Lett 129: 145-149.

22. Tominaga K, Saito Y, Mori K, Miyazawa N, Yokoi K, et al. (1992) An evaluation of serum microelement concentrations in lung cancer and matched non-cancer patients to determine the risk of developing lung cancer: a preliminary study. Jpn J Clin Oncol 22: 96-101.

23. Kune GA, Kune S, Watson LF, Pierce R, Field B, et al. (1989) Serum levels of beta-carotene, vitamin A, and zinc in male lung cancer cases and controls. Nutr Cancer 12: 169-176.

24. Salonen JT, Salonen R, Lappetelainen R, Maenpaa PH, Alfthan G, et al. (1985) Risk of cancer in relation to serum concentrations of selenium and vitamins A nd E: matched case-control analysis of prospective data. Br med J (Clin Res Ed) 290: 417-420.

25. Hinds MW, Kolonel LN, Hankin JH, Lee J (1984) Dietary vitamin A, carotene, vitamin C and risk oflung cancer in Hawaii. Am J Epidemiol 119: 227-237.

26. Wald N, Idle M, Boreham J, Bailey A (1980) Low serum-vitamin A and subsequent risk of cancer. Preliminary results of a prospective study. Lancet 2: 813-815.

27. Knekt P, Aromaa A, Maatela J, Aaran RK, Nikkari T, et al. (1990) Serum vitamin A and subsequent risk of cancer: cancer incidence follow-up of the Finnish Mobile Clinic Health Examination Survey. Am J Epidemiol 132: 857-870.

28. Ito Y, Wakai K, Suzuki K, Ozasa K, Watanabe Y, et al. (2005) Lung cancer mortality and serum levels of carotenoids, retinol, tocopherols, and folic acid in mena nd women: a case-control study nested in the JACC Study. J Epidemiol 15 Suppl 2: S140-149.

29. Ito Y, Wakai K, Suzuki K, Tamakoshi A, Seki N, et al. (2003) Serum carotenoids and mortality from lung cancer: a case-control study nested in the Japan Collaborative Cohort (JACC) study. Cancer Sci 94: 57-63.

30. LeGardeur BY, Lopez A, Jonson WD (1990) A case-control study of serum vitamins A, E, and C in lung cancer patients. Nutr Cancer 14: 133-140.

31. Connett JE, Kuller LH, Kjelsberg MO, Polk BF, Collins G, et al. (1989) Relationship between carotenoids and cancer. The Multiple Risk Factor Intervention Trial (MRFIT) Study. Cancer 64: 126-134.

32. Pastorino U, Pisani P, Berrino F, Andreoli C, Barbieri A, et al. (1987) Vitamin A and female lung cancer: a case-control study on plasma and diet. Nutr Cancer 10: 171-179.

33. Friedman GD, Blaner WS, Goodman DS, Vogelman JH, Brind JL, et al. (1986) Serum retinol and retinol-binding protein levels do not predict subsequent lung cancer. Am J Epidemiol 123: 781-789.

34. Menkes MS, Comstock GW, Vuilleumier JP, Helsing KJ, Rider Aa, et al. (1986) Serum beta-carotene, vitamins A and E, selenium, and the risk of lung cancer. N Engl J med 315: 1250-1254.

35. Darby S, Whitley E, Doll R, Key T, Silcocks P (2001) Diet, smoking and lung cancer: a case-control study of 1000 cases and 1500 controls in South-West England. Br J Cancer 84: 728-735.

36. Goodman GE, Thornquist M, Kestin M, Metch B, Anderson G, et al. (1996) The association between participant characteristics and serum concentrations of beta-carotene, retinol, reninyl palmitate, and alpha tocopherol among participants in the Carotene and Retinol Efficacy Trial (CARET) for prevention of lung cancer. Cancer Epidemiol Biomarkers Prev 5: 815-821.

37. Emri S, Kilickap S, Kadilar C, Khalil M, Akay H, et al. (2003) P-510 Serum vitamin E, vitamin C, beta carotene, and retinol levels and the risk of malignant pleural mesothelioma in Turkey. Lung Cancer 41: S219.

38. Sawicki J, Ostrowski J, Swietochowska B, Janik P, Sikora J, et al. (1985) Vitamin A (retinol) level in colon and lung cancer patient sera. Neoplasma 32: 225-227.

39. Jain NC, Gupta AP, Saksena HC (1984) Serum vitamin ‘A’ in bronchogenic carcinoma. J Assoc Physicians India 32: 485-486.

40. Atukorala S, Basu TK, Dickerson JW, Donaldson D, Sakula A (1979) Vitamin A, zinc and lung cancer. Br J Cancer 40: 927-931.

41. Basu TK, Donaldson D, Jenner M, Williams DC, Sakula A (1976) Plasma vitamin A in patients with bronchial carcinoma. Br J Cancer 33: 119-121.

42. Cohen MH, Primack A, Broder LE, Williams LR (1978) Vitamin A serum and dietary vitamin A intake in lung cancer patients. Cancer Lett 4: 51-54.

43. Moulas AN, Gerogianni IC, Papadopoulos D, Gourgoulianis KI (2006) Serum retinoic acid, retinol and retinyl palmitate levels in patients with lung cancer. Respirology 11: 169-174.

44. Gackowski D, Kowalewski J, Siomek A, Olinski R (2005) Oxidative DNA damage and antioxidant vitamin level: comparison among lung cancer patients, healthy smokers and nonsmokers. Int J Cancer 114: 153-156.

45. Mura P, Castel O, Gros N, Suire R (1987) Vitamin A concentrations in lung cancer. Clin Chem 33: 716.
