# Supplementary material for: Vitamin A and Retinoid Derivatives for Lung Cancer: A Systematic Review and Meta Analysis
Source: PLoS One. 2011 Jun 27;6(6):e21107. doi: 10.1371/journal.pone.0021107 (PMC3124481; doi:10.1371/journal.pone.0021107)
Supplement: Table S6 — Combination Effect, in vitro , Between Forms of Vitamin A and Chemotherapy Drugs. (DOC) [file pone.0021107.s006.doc]

**Table S6. Combination Effect, *in vitro*, Between Forms of Vitamin A and Chemotherapy Drugs**

|  | **Chemotherapy** | **CARBO** | **CDDP** | **CTX** | **DEX** | **DOX** | **DOC** | **GCB** | **PAX** | **VCR** | **VIN** | **VP-16** |
| --- | --- | --- | --- | --- | --- | --- | --- | --- | --- | --- | --- | --- |
| **Cell Line** |  |  |  |  |  |  |  |  |  |  |  |  |
| **Brain** |  |  |  |  |  |  |  |  |  |  |  |  |
| Medulloblastoma | |  | ATRA /+ [1] |  |  |  |  |  |  |  |  |  |
| **Glioblastoma** | |  |  |  |  |  |  |  |  |  |  |  |
|  | T98G |  |  |  |  |  |  |  | ATRA /+ [2]  13CRA /+ [2] |  |  |  |
|  | GB |  |  |  |  |  |  |  |  | RA /+ [3] |  |  |
| **Breast** |  |  |  |  |  |  |  |  |  |  |  |  |
|  | NMU-417 |  |  |  |  |  |  |  | Bex /+  [4] |  |  |  |
|  | MCF-7 |  | ATRA /+ [5]  Fen /± [5] |  |  | ATRA /+ [6,7] | 9CRA /↑↑ [8] |  |  |  |  |  |
|  | MCF-7/adr |  |  |  |  |  | 9CRA /↑↑ [8] |  |  |  |  |  |
|  | MDA-MB-231 |  |  |  |  |  |  |  | Bex /+ [9] |  |  |  |
|  | BT474 |  | ATRA /+ [5]  Fen /± [5] |  |  |  |  |  |  |  |  |  |
|  | SK-BR-3 |  | ATRA /+ [168]  Fen /± [5] |  |  |  |  |  |  |  |  |  |
|  | Mouse mammary |  |  |  | RP /+ [10] |  |  |  |  |  |  |  |
| **Cervical** |  |  |  |  |  |  |  |  |  |  |  |  |
|  | SiHa |  | 9CRA & radiation /+ [11]  Fen & radiation /+ [11] |  |  |  |  |  |  |  |  |  |
| **Head and Neck** | |  |  |  |  |  |  |  |  |  |  |  |
|  | UMSCC10b |  | ATRA /+ [12] |  |  |  |  |  |  |  |  |  |
|  | DDP resistant  UMSCC10b-Pt/15S |  | ATRA /+ [12] |  |  |  |  |  |  |  |  |  |
| **Leukemia** | |  |  |  |  |  |  |  |  |  |  |  |
|  | P388 |  | RP /+ [13] |  |  |  |  |  |  |  |  |  |
|  | HL60 |  |  |  |  | ATRA /↓ [14] |  |  | ATRA /↓ [14] |  |  | ATRA /↓ [15] |
|  | NB4 |  |  |  |  | ATRA /↓ [14]  ATRA /↓ [16] |  |  | ATRA /↓ [14] |  |  | ATRA /↓ [15] |
| **Melanoma** | |  |  |  |  |  |  |  |  |  |  |  |
|  | B16-F10s |  |  |  | ATRA /+ [17]  13CRA /+ [17]  9CRA /+ [17] |  |  |  |  |  |  |  |
| **Multiple Myeloma** | |  |  |  |  |  |  |  |  |  |  |  |
|  | U266 |  |  |  | ATRA /↑↑ [18]  13CRA /↑↑ [18]  9CRA /↑ [18] |  |  |  |  |  |  |  |
|  | RPMI 8226 |  |  |  | ATRA /↑↑ [18,19]  13CRA /↑ [18,19]  9CRA /↑↑ [18,19] |  |  |  |  |  |  |  |
|  | ATCC CRL 1646 FO |  |  |  | ATRA /+ [20] |  |  |  |  |  |  |  |
|  | OPM-2 |  |  |  | ATRA /+ [21] |  |  |  |  |  |  |  |
| **Neuroblastoma** | |  |  |  |  |  |  |  |  |  |  |  |
|  | SHSY5Y | Fen /↑↑ [22,23] | Fen /↑↑ [22,23] |  |  |  |  |  |  |  |  | Fen /↑↑ [22,23] |
| **Non Small cell Lung carcinoma** | |  |  |  |  |  |  |  |  |  |  |  |
|  | A427 |  |  |  |  |  |  |  | Bex /↑↑ [24] |  | Bex /↑↑ [24] |  |
|  | A549 |  |  | ATRA /+ [25]  13CRA /+ [25]  9CRA /+ [25] |  |  |  |  | Bex /↑↑ [24]  13CRA /↑↑ [26] |  | Bex /↑↑ [24] |  |
|  | Calu3 |  |  |  |  |  |  |  | Bex /↑↑ [24] |  | Bex /↑↑ [24] |  |
|  | EKVX |  |  |  |  |  |  |  | Bex /↑↑ [24] |  | Bex /↑↑ [24] |  |
|  | H322m |  |  |  |  |  |  |  | Bex /↑↑ [24] |  | Bex /↑↑ [24] |  |
|  | NCI-H460 |  | 13CRA /+ [26] |  |  |  |  |  | 13CRA /↑↑ [26] |  |  |  |
|  | H358m |  |  |  |  |  |  |  | Bex /↑↑ [24] |  | Bex /↑↑ [24] |  |
|  | H441 |  |  |  |  |  |  |  | Bex /↑↑ [24] |  | Bex /↑↑ [24] |  |
|  | HOP62 |  |  |  |  |  |  |  | Bex /↑↑ [24] |  | Bex /↑↑ [24] |  |
|  | HOP92 |  |  |  |  |  |  |  | Bex /↑↑ [24] |  | Bex /↑↑ [24] |  |
|  | SKMES1 |  |  |  |  |  |  |  | Bex /↑↑ [24] |  | Bex /↑↑ [24] |  |
| **Small Cell Lung Carinoma** | |  |  |  |  |  |  |  |  |  |  |  |
|  | NCI-H82 |  | Fen /↑ [27] |  |  |  |  |  | Fen /↓↓ [27] |  |  | Fen /↑ [27] |
|  | NCI-H446 |  | Fen /↑ [27] |  |  |  |  |  | Fen /↑ [27] |  |  | Fen /↑ [27] |
|  | NCI-H345 |  |  |  |  |  |  |  |  |  |  |  |
|  | NCI-H69 |  |  |  |  |  |  |  |  |  |  |  |
|  | SHP77 |  | 13CRA /↑ [26] |  |  |  |  |  | 13CRA /↑ [26] |  |  |  |
| **Osteosarcoma** | |  |  |  |  |  |  |  |  |  |  |  |
|  | HOS-8603 |  |  |  | ATRA /↑ [28] |  |  |  |  |  |  |  |
| **Ovarian** | |  |  |  |  |  |  |  |  |  |  |  |
|  | 2008 |  | ATRA /± [29] |  |  |  |  |  |  |  |  |  |
|  | 2008/C13 |  | ATRA /↑ [29] |  |  |  |  |  |  |  |  |  |
|  | OVCCR1 |  | ATRA /↑ [29] |  |  |  |  |  |  |  |  |  |
|  | IGROV-1 |  | ATRA /± [29] |  |  |  |  |  |  |  |  |  |
|  | NIHOVCAR3 |  | ATRA /↑ [29] |  |  |  |  |  |  |  |  |  |
|  | SKOV |  | ATRA /↑ [29] |  |  |  |  |  |  |  |  |  |
|  | A2780 |  | ATRA /± [29]  Fen /↑ [30] |  |  |  |  |  |  |  |  |  |
| **Pancreatic** | |  |  |  |  |  |  |  |  |  |  |  |
|  | T3M-4 |  | 9CRA /+ [31] |  |  |  |  | 9CRA /+ [31] |  |  |  |  |
|  | BxPc-3 |  | ATRA /+ [31]  9CRA /+ [31] |  |  |  |  | ATRA /+ [31]  9CRA /+ [31] |  |  |  |  |
| **Squamous Cell Carcinoma** | |  |  |  |  |  |  |  |  |  |  |  |
|  | SCC1483 |  | ATRA /↑↑ [32] |  |  |  |  |  |  |  |  |  |
|  | MDA686Ln |  | ATRA /↑↑ [32] |  |  |  |  |  |  |  |  |  |
|  | A431 |  | ATRA /↑↑ [32] |  |  |  |  |  |  |  |  |  |
| **Prostate** | |  |  |  |  |  |  |  |  |  |  |  |
|  | DU145 |  |  |  |  |  | CRA /↑ [33]  ATRA /↑↑ [34] |  |  |  |  |  |
|  | PC3 |  |  |  |  |  | CRA /↑ [33] |  |  |  |  |  |
|  | LnCaP |  |  |  |  |  | CRA /↑ [33]  ATRA /↑↑ [34] |  |  |  |  |  |
| **Other** | |  |  |  |  |  |  |  |  |  |  |  |
|  | Tera-2 |  | ATRA /+ [35] |  |  |  |  |  |  |  |  |  |
|  | Tera2-CP |  | ATRA /+ [35] |  |  |  |  |  |  |  |  |  |
|  | PCC4 |  | ATRA /+ [36] |  |  |  |  |  |  |  |  | ATRA /+ [36] |

| ↑↑ | Synergy exists where the Combination Index (CI)i s < 1. In the article, where the authors calculated the CI and the CI was found to be less than 1. |
| --- | --- |
| **↑** | Additive effect exists where the Combination Index =1. In the article, where the authors calculated the CI and the CI was found to be equal to 1. |
| + | Positive impact – where the use of the two agents in series or combination resulted in increased growth inhibition that was greater than the single agent alone. |
| ± | Slight antagonism exists where the Combination Index (CI) is just above 1. |
| ↓ | Negative impact where the use of the two agents in series or combination resulted in decreased growth inhibition versus the drug agent. |
| ↓↓ | Antagonism exists where the Combination Index (CI) is greater than 1. |
| **Abbreviations**: **9CRA** 9 *cis* Retinoic acid; **13CRA** 13 *cis* Retinoic acid; **ATRA** all trans Retinoic Acid; **Bex** Bexarotene; **CARBO** Carboplatin; **CDDP** Cisplatin; **CTX** Cyclophosphamide; **DEX** Dexamethasone; **DOC** Docetaxel; **DOX** Doxorubicin; **Fen** Fenretinide; **GCB** Gemcitibine; **PAX** Pacitaxel; **RA** Retinoic Acid; **RP** Retinol palmitate; **VCR** Vincristine; **VIN** Vinorelbine; **VP-16** Etoposide. | |

**References**

1. Liu J, Guo L, Jun-Wei L, Liu N, Li H (2000). All-trans retinoic acid modulated fas expression and enhances chemosensitivity of human medulloblastoma cells. Int J Mol Med 5: 145-149.

2. Das A, Banik NL, Ray SK (2008) Retinoids induced astrocytic differentiation with down regulation of telomerase activity and enhanced sensitivity to taxol for apoptosis in human glioblastoma T98G and U87MG cells. J Neurooncol 87: 9-22.

3. Tani E, Morimura T, Kaba K, Itagaki T (1980) Preliminary study of the effects of vitamin A on antineoplastic activities of chemotherapeutic agents in glioma. Neurol Med Chir (Tokyo) 20: 665-677.

4. Yen WC, Predente RY, Lamph WW (2004) Synergistic effect of a retinoid X receptor-selective ligand bexarotene (LGD1069, Targretin) and paclitaxel (Taxol) in mammary carcinoma. Breast Cancer Res Treat 88: 141-148.

5. Grunt ThW, Dittrich E, Offterdinger M, Scheider SM, Dittrich Ch, et al. (1998) Effects of retinoic acid and fenretinide on the c-erbB-2 expression, growth and cisplatin sensitivity of breast cancer cells. Br J Cancer 78: 79-87.

6. Czeczuga-Sememiuk E, Wolczynski S, Dabrawska M, Dzieciol J, Anchimj T (2004) The effect of doxorubicin and retinoids on proliferation, necrosis and apoptosis in MCF-7 breast cancer cells. Folia Histochem Cytobiol 42: 221-227.

7. Toma S, Maselli G, Dastoli G, De Francisci E, Raffo P (1997) Synergistic effect between doxorubicin and a low dose of all-trans-retinoic acid in MCF-7 breast cancer cell line. Cancer Lett 116: 103-110.

8. Budman DR, Calabro A (2002) In vitro search for synergy and antagonism: evaluation of docetaxel combinations in breast cancer cell lines. Breast Cancer Res Treat 74: 41-46.

9. Yen, WC, Lamph WW (2005) The selective retinoid X receptor agonist bexarotene (LGD1069, Targretin) prevents and overcomes multidrug resistance in advanced breast carcinoma. Mol Cancer Ther 4: 824-834.

10. Rieber M, Seyler L (1980) Growth inhibition of mouse mammary tumor cells by dexamethasone concurrent with enhanced endogenous protein phosphorylation: effects of retinoic acid. Cell Biol Int Rep 4: 1075-1079.

11. Scribner Jr DR, Benbrook DM (2002) Retinoids enhance cisplatin-based chemoradiation in cervical cancer cells in vitro. Gynecol Oncol 85: 223-225.

12. Aebi S, Kroning R, Cenni B, Sharma A, Fink D, et al. (1997) All-trans retinoic acid enhances cisplatin-induced apoptosis in human ovarian adenocarcinoma and in squamous head and neck cancer cells. Clin Cancer Res 76: 887-894.

13. Nakagawa M, Yamaguchi T, Ueda H, Shiraishi N, Komiyama S, et al. (1985) Potentiation by vitamin A of the action of anticancer agents against murine tumors. Jpn J Cancer Res 76: 887-894.

14. Xia L, Wurmbach E, Waxman S, Jing Y (2006) Upregulation of Bfl-1/ A1 in leukemia cells undergoing differentiation by all-trans retinoic acid treatment attenuates chemotherapeutic agent-induced apoptosis. Leukemia 20: 1009-1016.

15. Jasek E, Mirecka J, Litwin JA (2008) Effect of differentiating agents (all-trans retinoic acid and phorbol 12-myristate 13-acetate) on drug sensitivity of HL60 and NB4 cells in vitro. Folia Histochem Cytobiol 46: 323-330.

16. Tabe Y, Konopleva M, Contractor R, Munsell M, Schober WD, et al. (2006) Upregulation of MDR1 and induction of doxorubicin resistance by histone deacetylase inhibitor depsipeptide (FK228) and ATRA in actue promyelocytic leukemia cells. Blood 107: 1546-1554.

17. Liu X, Chan SY, Ho PCL (2008) Comparison of the in vitro and in vivo effects of retinoids either alone or in combination with cisplatin and 5-fluorouracil on tumor development and metastasis of melanoma. Cancer Chemother Pharmacol 63: 167-174.

18. Tosi P, Pellacani A, Visani G, Ottaviani E, Ronconi S, et al. (1999) In vitro treatment with retinoids decreases bcl-2 protein expression and enhances dexamethasone-induced cytotoxicity and apoptosis in multiple myeloma cells. Eur J Haematol 62: 143-148.

19. Smith MR, Xie T, Joshi I, Schilder RJ (1998) Dexamethasone plus retinoids decrease IL-6/IL-6 receptor and induced apoptosis in myeloma cells. Br J Haematol 102: 1090-1097.

20. Ozdemir F, Esen N, Ovali E, Tekelioglu Y, Yilmaz M, et al. (2004) Effects of dexamethasone, all-trans retinoic acid, vitamin D3 and interferon-alpha on FO myeloma cells. Chemotherapy 50: 190-193.

21. Chen YH, Desai P, Shiao RT, Lavelle D, Haleem A, et al. (1996) Inhibition of myeloma cell growth by dexamethasone and all-trans retinoic acid: synergy through modulation of interleukin-6 autocrine loop at multiple sites. Blood 87: 314-323.

22. Lovat PE, Ranalli M, Bernassola F, Tilby M, Malcolm AJ, et al. (2000) Synergistic induction of apoptosis of neuroblastoma by fenretinide or CD437 in combination with chemotherapeutic drugs. Int J Cancer 88: 977-985.

23. Corazzari M, Lovat PE, Oliverio S, Pearson ADJ, Piacentini M, et al. (2003) Growth and DNA damage-inducible transcription factor 153 mediates apoptosis in response to fenretinide by not synergy between fenretinide and chemotherapeutic drugs in neuroblastoma. Mol Pharmacol 64: 1370-1378.

24. Hermann TW, Yen W-C, Tooker P, Fan B, Roegner K, et al. (2005) The retinoid X receptor agonist bexarotene (Targretin) synergistically enhances the growth inhibitory activity of cytotoxic drugs in non-small cell lung cancer cells. Lung Cancer 50: 9-18.

25. Moreb JS, Gabr A, Bartikar GR, Gowda S, Zucali JR, et al. (2005) Retinoic acid down-regulates aldehyde dehydrogenase and increases cytotoxicity of 4-hydroperoxycyclophosphamide and acetaldehyde. J Pharmacol Exp Ther 312: 339-345.

26. Soriano AF, Helfrich B, Chan DC, Heasley LE, Bunn PA, Jr, et al. (1999) Synergistic effects of new chemopreventive agents and conventional cytotoxic agents against human lung cancer cell lines. Cancer Research 59: 6178-6184.

27. Kalemkerian GP, Ou X (1999) Activity of fenretinide plus chemotherapeutic agents in small-cell lung cancer cell lines. Cancer Chemotherapy and Pharmacology 43: 145-150.

28. Song LN (1994) Effects of retinoic acid and dexamethasone on proliferation, differentiation, and glucocorticoid receptor expression in cultured human osteosarcoma cells. Oncol Res 6: 111-118.

29. Caliaro MJ, Vitaux P, Lafon C, Lochon I, Nehme A, et al. (1997) Multifactorial mechanism for the poteniation of cisplatin (CDDP) cytotxicity by all-trans retinoic acid (ATRA) in human ovarian carcinoma cell lines. Br J Cancer 75: 333-340.

30. Supino R, Crosti M, Clerici M, Warlters A, Cleris L, et al. (1996) Induction of apoptosis by fenretinide (4HPR) in human ovarian carcinoma cells and its association with retinoic acid receptor expression. Int J Cancer 65: 491-497.

31. Pettersson F, Colston KW, Dalgleish AG (2001) Retinoic acid enhances the cytotoxic effects of gemcitabine and cisplatin in pancreatic adenocarcinoma cells. Pancreas 23: 273-279.

32. Sacks PG, Harris D, Chou TC (1995) Modulation of growth and prolifeation in squamous cell carcinoma by retinoic acid: a rational for combination therapy with chemotherapeutic agents. Int J Cancer 61: 409-415.

33. Budman DR, Calabro A, Kreis W (2002) Synergistic and antagonistic combinations of drugs in human prostate cancer cell lines in vitro. Anti-Cancer Drugs 13: 1011-1016.

34. Nehme A, Varadarajan P, Sellakumar G, Gerhold M, Niedner H, et al. (2001) Modulation of docetaxel-induced apoptosis and cell cycle arrest by all-trans retinoic acid in prostate cancer cells. Br J Cancer 84: 1571-1576.

35. Timmer-Bosscha H, De Vries EGE, Meijer C, Oosterhuis JW, Mulder NH (1998) Differential effects of all-trans-retinoic acid, docosahexaenoic acid, and hexadecylphosphocholine on cisplain-induced cytotoxicity and apoptosis in a cisplatin-sensitive and resistance human embryonal carcinoma cell line. Cancer Chemother Pharmacol 41: 469-476.

36. Guchelaar HG, Timmer-Bosscha H, Dam-Meiring A, Uges DR, Oosterhuis JW, et al. (1993) Enhancement of cisplatin and etoposide cytotoxicity after all-trans retinoic-acid-induced cellular differentiation of a murine embryonal carcinoma cell line. Int J Cancer 55: 442-447.
